# Synergistic Integration of Fe–N_4_ Single‐Atom Sites and Directionally Aligned Electrode Architecture for High‐Performance Lithium–Sulfur Batteries

**DOI:** 10.1002/advs.77768

**Published:** 2026-09-11

**Authors:** Lin Shen, Yijia Zou, Yuming Wang, Erya Gao, Christopher S. Allen, Tianyi Zhang, Stephen J. Skinner, Nigel P. Brandon, Chun Huang

**Affiliations:** ^1^ Department of Materials Imperial College London London UK; ^2^ Electron Physical Science Imaging Centre (ePSIC) Diamond Light Source Didcot UK; ^3^ Department of Earth Science and Engineering Imperial College London London UK; ^4^ The Faraday Institution Didcot UK; ^5^ Research Complex at Harwell Harwell Campus Didcot UK

**Keywords:** directional ice templating, lithium‐sulfur batteries, multi‐length scale structure, single‐atom catalysts, sulfur redox kinetics

## Abstract

Sluggish kinetics of redox reactions and slow ion transport restrict the performance of lithium‐sulfur (Li─S) batteries. Here, we fabricate cathodes containing vertically aligned porous structures hosting atomically dispersed Fe─N_4_ single‐atom catalyst (SAC) sites on graphitic carbon nitride (g‐C_3_N_4_). Density functional theory (DFT) calculations suggest that the Fe─N_4_ sites strengthen Li_2_S adsorption, optimize electronic structure, and lower the reaction energy change associated with liquid‐solid transition and Li_2_S oxidation. Experimental results demonstrate that the atomically dispersed Fe─N_4_ sites in the vertically aligned porous cathodes made by directional ice templating (DIT) accelerate Li^+^ ion transport and enable high sulfur loading while exposing abundant catalytic centers, resulting in strong polysulfide affinity, promoted nucleation and decomposition of Li_2_S bidirectional redox catalysis, and suppressed polysulfide shuttle effect. Benefiting from this structural‐catalytic synergy, the cathode delivers a high capacity of 1299.2 mAh g^−1^ at 0.1 C, and the capacity is retained at 505.9 mAh g^−1^ after 1 000 cycles at 0.5 C, with a low capacity decay rate of ∼0.042% per cycle. This study highlights a scalable strategy to integrate SAC with vertically aligned porous electrode architecture that promotes fast kinetics of sulfur redox in both directions and mass transport for Li─S batteries.

## Graphical abstract

A directionally ice‐templated sulfur cathode integrated with atomically dispersed Fe–N_4_ catalytic sites is developed for high‐performance lithium–sulfur batteries. The structure–catalysis synergy accelerates Li^+^ transport and polysulfide conversion, leading to high sulfur utilization and stable long‐term cycling performance.

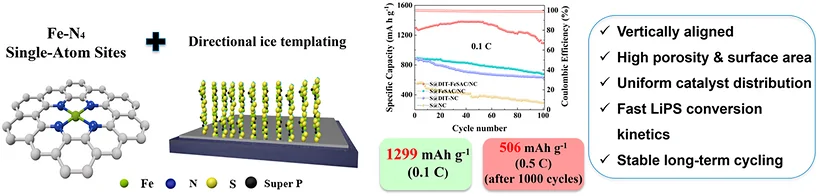

## 1 Introduction

High energy density energy storage systems have attracted significant attention for electric transportation and grid‐scale storage of intermittent renewable energy in the transition to net‐zero [1, 2, 3, 4, 5, 6, 7]. Compared to traditional lithium‐ion batteries, lithium‐sulfur (Li─S) batteries offer advantages such as high theoretical energy density (2600 Wh kg^−1^) and capacity (1675 mAh g^−1^), abundant cathode material reserves, low cost, and environmental friendliness [8, 9], making them promising next‐generation energy storage technologies [10, 11]. However, the practical application of Li─S batteries is currently hindered by several critical scientific challenges, including the “shuttle effect” of soluble lithium polysulfides (LiPSs, Li_2_S_n_, 4 ≤ n ≤ 8), the low electrical conductivity of sulfur and discharge products (Li_2_S_2_/Li_2_S), and volume expansion during discharge [12]. The diffusion of soluble LiPS from the sulfur cathode to the lithium anode leads to the formation of solid‐state Li_2_S_2_/Li_2_S, resulting in irreversible loss of active sulfur, reduced Coulombic efficiency, and degraded performance [13, 14]. Consequently, designing cathode materials and nanostructures that enhance LiPS catalytic conversion efficiency, improve sulfur utilization, and mitigate the shuttle effect is critical to improve energy storage performance and reduce Li─S battery degradation [15].

Single‐atom catalysts (SACs), which provide isolated metal atomic sites on substrates, have garnered significant attention [16, 17]. Compared to nanoclusters or nanoparticles, single atoms offer the highest theoretical atom utilization efficiency, tunable electronic structures, and distinct surface properties [18]. SACs show significant potential in accelerating reaction kinetics and improving the utilization of active materials [19]. To date, several single‐atom catalysts with metal‐N_x_ active sites have been employed as electrocatalytic centers for LiPS adsorption and conversion. For instance, Fe─N_2_ single atoms on a C_3_N_4_ substrate using marcroporous diatomite as a template provided robust catalytic activity, enhancing the sulfur redox reactions [20]. Theoretical and experimental results showed that Ni─N_4_ structures inhibit the reaction between Ni and S_8_, preventing passivation, and batteries with Ni─N_4_ single atoms exhibited a high capacity of 604.1 mAh g^−^^1^ at 3C [21]. Theoretical calculations revealed that asymmetric single‐atom configurations with P and S dual coordination promoted d‐p orbital hybridization between Co and S while retaining more 3d orbital electrons on Co atoms, thereby enhancing adsorption capacity and catalytic activity [22].

Directional ice templating (DIT) has gained attention for its ability to create ordered porous structures that provide enhanced pathways for Li^+^ transport [23, 24]. Conventional electrode processing methodology suspends a mixture of electrode materials in a solvent and coats the viscous electrode suspension onto a current collector. The wet electrodes undergo heating, where the solvent is evaporated, resulting in randomly porous structures that restrict ion diffusion [25]. In contrast, during the DIT process, a freezing temperature gradient is applied to the electrode suspension from the bottom of the suspension. Ice crystals start to nucleate at the supercooling interface, followed by vertical growth of ice dendrites upwards along the temperature gradient, pushing the electrode material particles in between the ice dendrites [26]. Ice is then sublimed; the self‐assembled electrode nanostructure exhibits vertically aligned, interleaving columns of electrode material particles and pore arrays, where the material and pore channel diameters are controlled by the freezing temperature gradient and ice solidification kinetics [27]. The anisotropic DIT nanostructure reduces both internal electrical resistance and ion transport impedance, as well as increasing electrode/electrolyte interfacial surface area to increase the utilization efficiency of the electrode active materials [26, 28].

Herein, we present a synergistic design for Li─S batteries by integrating atomically dispersed Fe─N_4_ active sites with a DIT porous architecture. Fe was selected as the catalytic center because its moderate d‐electron configuration provides an optimal balance between polysulfide adsorption and conversion through appropriate d–p orbital hybridization with sulfur intermediates, avoiding both insufficient anchoring and excessive surface passivation [29]. Meanwhile, the vertically aligned DIT architecture creates low‐tortuosity ion‐transport pathways, improves electrolyte penetration, and maximizes the accessibility of Fe─N_4_ catalytic sites. As a result, the DIT framework and Fe─N_4_ single‐atom sites work synergistically to accelerate sulfur redox kinetics and Li^+^ transport. Benefiting from this structural–catalytic synergy, the S@DIT‐FeSAC/NC cathode delivers an initial discharge capacity of 1299.2 mAh g^−^^1^ at 0.1 C and retains 505.9 mAh g^−^^1^ after 1000 cycles at 0.5 C, corresponding to an average capacity decay rate of only 0.042% per cycle. Although SACs can accelerate polysulfide adsorption and conversion, their catalytic activity is still limited by sluggish ion transport and restricted accessibility of active sites in conventional randomly porous electrodes. Conversely, directionally structured electrodes facilitate ion transport, but little work has been performed to intrinsically accelerate sulfur redox reactions and develop scaling up of processing to make vertically aligned electrode structures. The novelty of this work lies in integrating atomically dispersed catalytic sites with a directionally aligned electrode architecture to simultaneously address both sulfur redox kinetics and mass transport challenges that cannot be addressed by catalyst or electrode architecture alone. We have also developed a scalable processing instrument to make the directionally structured electrodes. This work demonstrates a scalable strategy that couples atomically dispersed catalytic sites with engineered ion transport architectures, providing a promising route toward high‐energy‐density and long‐lifespan Li─S batteries.

## 2 Results and Discussion

**FIGURE 1:**
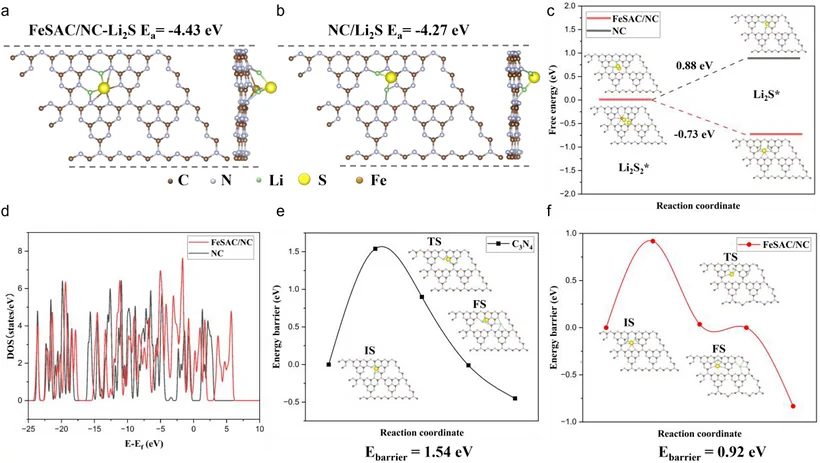
Optimized configurations of Li_2_S on (a) FeSAC/NC and (b) NC. (c) Reaction energy change profile for Li_2_S oxidation on FeSAC/NC and NC. (d) DOS of FeSAC/NC and NC. (e) Reaction energy profile on NC. (f) Reaction energy profile on FeSAC/NC.

Density functional theory (DFT) calculations were performed to systematically compare the catalytic behavior of Fe SAC on g‐C_3_N_4_ (FeSAC/NC) and g‐C_3_N_4_‐only (NC) toward LiPS adsorption and conversion, aiming to elucidate the multiple synergistic roles of Fe SAC in the cathode reactions. Figure 1a,b presents the optimized adsorption configurations of Li_2_S on FeSAC/NC and NC surfaces. The calculated adsorption energies were −4.43 eV for FeSAC/NC and −4.27 eV for NC, indicating moderately more favorable Li_2_S adsorption on the Fe─N_4_‐containing surface. The calculations focused on studying the effect of the Fe─N_4_ sites on the critical Li_2_S_2_‐to‐Li_2_S terminal conversion, as Li_2_S_2_‐to‐Li_2_S exhibits the largest positive reaction energy change amongst the successive reduction steps [30]. FeSAC/NC exhibited a lower reaction energy change for the Li_2_S_2_‐to‐Li_2_S conversion than NC, indicating that the reduction toward Li_2_S formation was thermodynamically more favorable on the Fe─N_4_ sites (Figure 1c) [31, 32]. This thermodynamic preference may favor Li_2_S formation and precipitation but does not directly imply a reduced kinetic nucleation barrier [20, 33]. The DOS analysis revealed modified electronic‐state contributions near the Fermi level in FeSAC/NC (Figure 1d). PDOS analysis (Figures S1–S3) further identifies that Fe 3d‐derived states contribute to the electronic structure in this energy region surrounding the Fermi level. These results indicate that incorporation of the Fe─N_4_ site modifies the near‐Fermi electronic structure of the NC framework. Notably, the Li_2_S decomposition reaction energy change on FeSAC/NC (∼0.92 eV) was lower than that on NC (∼1.54 eV), indicating that Li_2_S oxidation was thermodynamically more favorable on the Fe─N_4_‐containing surface (Figure 1e,f). Collectively, these calculations suggested that FeSAC/NC provided moderately more favorable Li_2_S adsorption, modified the electronic structure near the Fermi level, and promoted thermodynamically favorable sulfur conversion [34].

**FIGURE 2:**
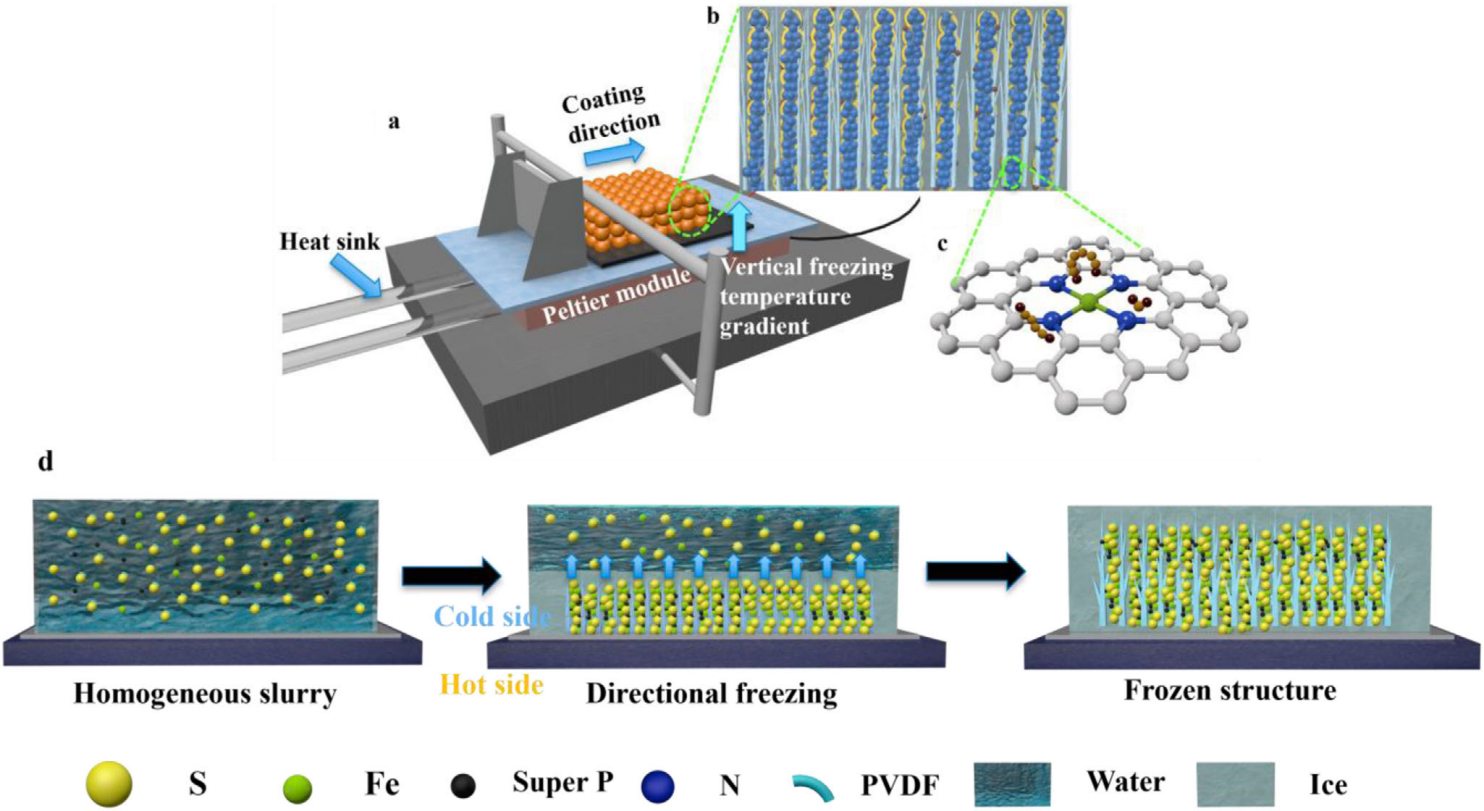
Schematic diagrams illustrating (a) the scalable directional ice templating (DIT) instrument developed in‐house where a vertical freezing temperature gradient is applied from the Peltier module supercooling interface; (b) magnified cathode structure showing vertically aligned ice column growth that pushes electrode materials in between to form vertical columns; (c) magnified cathode material FeSAC/NC showing Fe─N_4_ single‐atom catalytic sites on graphitic carbon nitride g‐C_3_N_4_; (d) formation mechanism of the DIT process.

Figure 2a illustrates the scalable DIT instrument that we have built for the self‐assembly methodology of making the electrodes containing vertically aligned pore arrays (Figure 2b) containing the cathode material FeSAC/NC (Figure 2c). A Peltier module was used to establish a vertical freezing temperature gradient, causing the suspension to freeze directionally from the current collector side. As shown in Figure 2d, ice crystals nucleate at the supercooling interface next to the cold side first, and the temperature difference between the supercooling interface and ambient conditions above the wet electrode creates a vertical temperature gradient that guides the vertical direction of ice column growth, promoting the growth of a columnar ice structure perpendicular to the cold side while pushing the electrode materials in between the ice columns. Finally, the ice is sublimed, resulting in cathode structures containing vertically aligned pore arrays; we call these cathodes S@DIT‐FeSAC/NC and S@DIT‐NC (without FeSAC). Cathodes containing the same materials made by conventional slurry coating without applying the temperature gradient during fabrication (S@FeSAC/NC) and cathodes without FeSAC made by slurry coating (S@NC) were also fabricated for comparison. A photograph of the actual directional ice‐templating system is presented in Figure S4, where the freezing unit was added to the coating bed of a commercial coater to increase scalability and facilitate practical fabrication.

**FIGURE 3:**
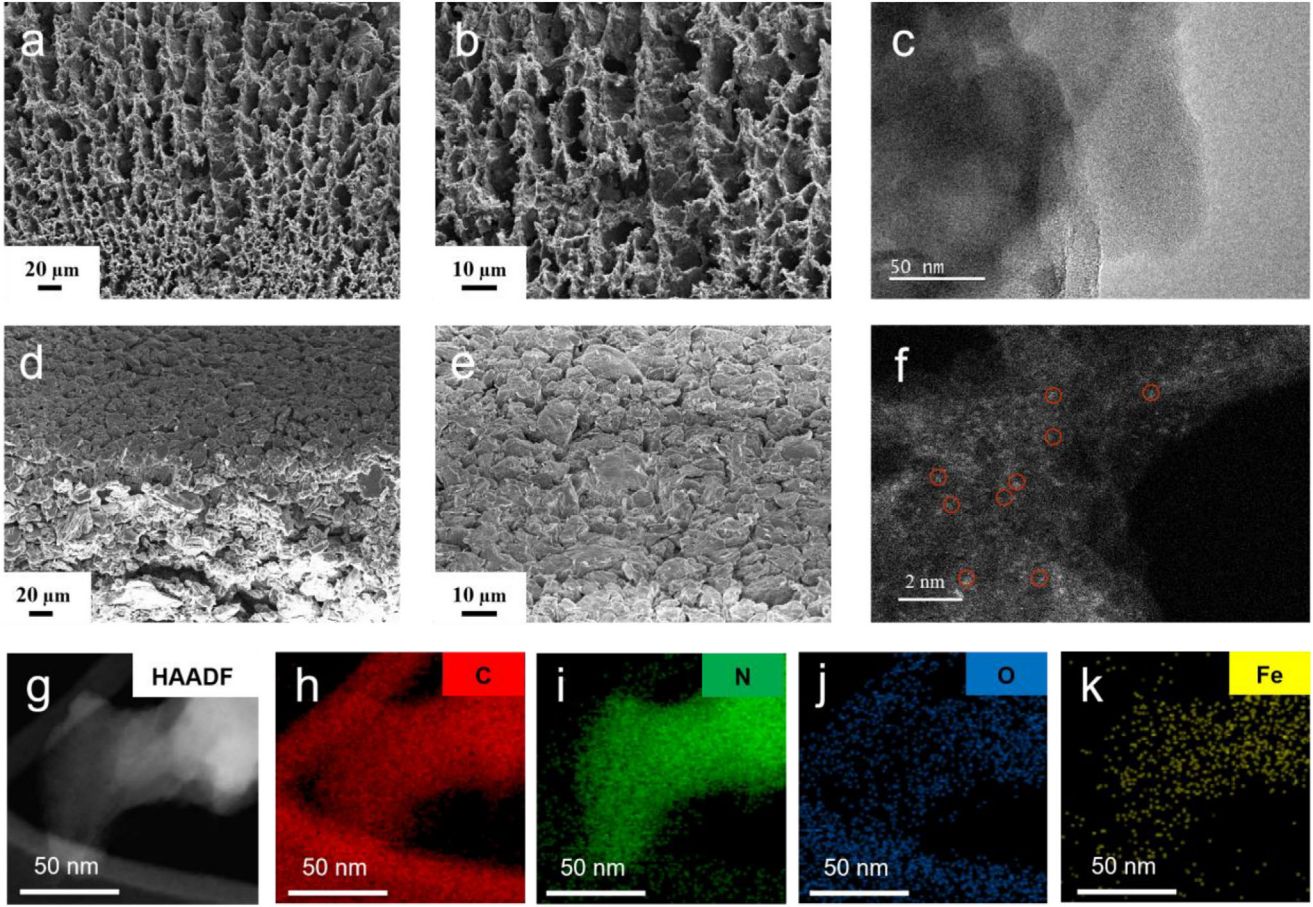
(a) Cross‐sectional SEM image of the S@DIT‐FeSAC/NC cathode; and magnified in (b). (c) TEM image of FeSAC/NC. (d) Cross‐sectional SEM image of the S@NC cathode; and magnified in (e). (f) HAADF‐STEM image of FeSAC/NC. (g) Smaller field‐of‐view HAADF‐STEM image of FeSAC/NC where elemental mapping is taken; and (h–k) Elemental maps of C, N, O, Fe.

Figure 3a,b show the cross‐sectional scanning electron microscope (SEM) images of S@DIT‐FeSAC/NC, indicating that the structure features vertically aligned porous channels. In contrast, electrodes prepared using the conventional slurry coating method with the same mass loading exhibited closed pores (Figure 3d,e) [35]. The pore size distributions of the S@DIT‐FeSAC/NC cathode and S@NC cathode made by slurry coating were calculated from cross‐sectional SEM images. As shown in Figure S5, the S@NC cathode exhibited a broad pore size distribution with a main peak at 1–3 µm and a noticeable fraction of larger pores of about 8 µm. In contrast, the S@DIT‐FeSAC/NC electrode showed a narrow distribution, with pore sizes concentrated in the 1‐3 µm range, indicating a higher proportion of small pores. This more uniform pore structure provided a homogeneous reaction interface for electrolyte infiltration and sulfur dispersion, which was beneficial for enhancing sulfur utilization and cycling stability. To quantitatively evaluate ion‐transport pathways, tortuosity analysis was performed based on segmented cross‐sectional SEM images (Figure S6). The DIT electrode exhibited a substantially lower tortuosity factor along the through‐thickness z direction (τ_z_ = 3.68) than the slurry‐coated electrode (τ_z_ = 14.4), indicating straighter and more continuous ion‐transport pathways. Figure S7 presents the x‐ray diffraction (XRD) patterns of FeSAC/NC and NC. The weak diffraction feature at approximately 13° was assigned to the (100) in‐plane structural packing of tri‐s‐triazine units in graphitic carbon nitride (g‐C_3_N_4_), while the diffraction peak at approximately 27° corresponded to the (002) interlayer stacking of g‐C_3_N_4_ [36, 37]. No diffraction peaks corresponding to crystalline Fe or Fe‐containing compounds were observed in the XRD pattern, as XRD is a bulk characterization technique and the atomically dispersed Fe species in FeSAC/NC lack long‐range translational symmetry. Without a periodic crystal lattice, the isolated species cannot satisfy the Bragg condition for diffraction [38]. Despite being a bulk characterization technique, the absence of Fe‐related peaks in the XRD pattern suggested the lack of crystalline Fe agglomerates above the detection limit, thereby supporting the highly dispersed nature of the Fe species. The structure of the obtained FeSAC/NC was further analyzed through transmission electron microscopy (TEM), as shown in Figure 3c. No clear lattice fringes were detected in FeSAC/NC. The corresponding energy‐dispersive x‐ray spectroscopy (EDS) analysis presented in Figure S8 identified the presence of C, N, and Fe. Aberration‐corrected high‐angle annular dark‐field scanning transmission electron microscopy (HAADF‐STEM) provided direct evidence for the presence of species on a substrate [39]. As displayed in Figure 3f and Figures S9 and S10a, the isolated bright features, highlighted by the red circles, were assigned to individual or small clusters of atoms which were relatively heavy with respect to the support (based on atomic number contrast, Z‐contrast). The intensity line scan (shown in Figure S10) also shows the significantly higher intensity at these individual bright features than the surrounding carbon matrix, which is consistent with the higher Z‐contrast of Fe atoms. Combined with EDS mapping of FeSAC/NC (Figure 3g–k), we interpret these bright features as Fe, and Fe, C, N, and O were uniformly distributed.

**FIGURE 4:**
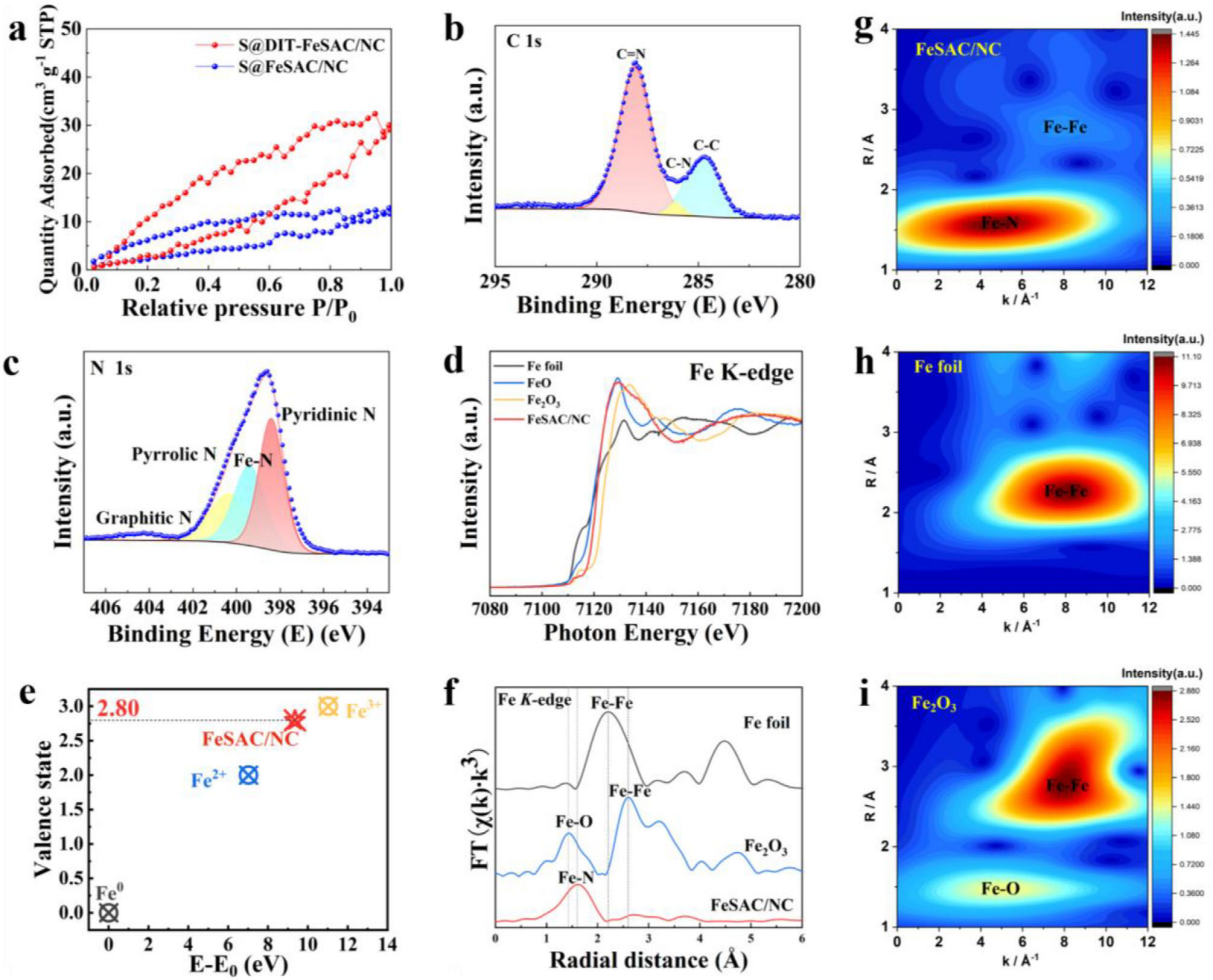
(a) N_2_ adsorption/desorption isotherms of S@DIT‐FeSAC/NC and S@FeSAC/NC electrodes. XPS: (b) C_1s_ spectrum and (c) N_1s_ spectrum of the FeSAC/NC material in powder form. (d) XANES of FeSAC/NC, Fe foil, FeO, and Fe_2_O_3_ in powder form. (e) Fe valence state estimation based on absorption edge energy shift. (f) FT‐EXAFS spectra of FeSAC/NC, Fe foil, and Fe_2_O_3_. Wavelet transform EXAFS of (g) FeSAC/NC; (h) Fe foil; and (i) Fe_2_O_3_.

N_2_ adsorption–desorption analysis was employed to examine the surface area and pore characteristics of S@DIT‐FeSAC/NC and S@FeSAC/NC electrodes (Figure 4a). The Brunauer‐Emmett‐Teller (BET) results revealed that the accessible surface area of the S@DIT‐FeSAC/NC and S@FeSAC/NC cathodes was 22.3 and 11.7 m^2^ g^−1^, respectively, and the S@DIT‐FeSAC/NC cathode showed a higher pore volume than the S@FeSAC/NC cathode [40]. Such porous architecture comprising vertically aligned macroporous channels promoted electrolyte infiltration, accommodated sulfur species, and facilitated ion transport. Thermogravimetric analysis (TGA) showed sulfur contents of 76.0 wt.% for S@FeSAC/NC powder and 78.1 wt.% for S@NC powder (Figure S11), indicating comparable sulfur loadings. X‐ray photoelectron spectroscopy (XPS) probed the surface valence states and chemical environment of the FeSAC/NC material [41]. The survey spectrum (Figure S12) confirmed the coexistence of Fe, N, O, and C elements in FeSAC/NC (in contrast to the absence of Fe in the survey spectrum of NC in Figure S13). The C_1s_ spectrum of FeSAC/NC (Figure 4b) revealed three peaks at 284.8, 286.2, and 288.2 eV, corresponding to C─C, C─N, and C═N bonds, respectively (similar features of the C_1s_ spectrum for NC in Figure S14). Figure 4c shows the N_1s_ spectrum of FeSAC/NC, which was deconvoluted into three types of nitrogen species: pyridinic‐N, Fe‐N, and pyrrolic‐N, with peaks located at 398.2, 399.5, and 400.4 eV, respectively [42]. These features of the C_1s_ and N_1s_ spectra of FeSAC/NC were characteristic of g‐C_3_N_4_ [20]. The XPS N_1s_ spectrum of the NC material (Figure S15) showed similar features but lacked the Fe‐N bond, confirming the absence of Fe in the NC material. In the Fe_2p_ spectrum of FeSAC/NC (Figure S16), two characteristic peaks at 710.0 and 723.2 eV corresponded to Fe 2p_3/2_ and Fe 2p_1/2_, respectively. The absence of the Fe^0^ signal at 706.7 eV indicated that it was unlikely for Fe on its own to be present in clusters [42]. Inductively Coupled Plasma Optical Emission Spectroscopy (ICP‐OES) analysis determined a Fe content of 1.08 ± 0.09 wt.% in the FeSAC/NC sample, confirming the successful incorporation of Fe species into the carbon framework (Figure S17).

To further elucidate the coordination configuration and oxidation state of Fe in FeSAC/NC, x‐ray absorption fine structure (XAFS) measurements were carried out. Figure 4d shows the Fe K‐edge x‐ray absorption near‐edge structure (XANES) spectra of FeSAC/NC, Fe foil, FeO and Fe_2_O_3_. Compared with Fe foil, the Fe K‐edge of FeSAC/NC was shifted to higher photon energy compared to the metallic Fe foil, situating between FeO and Fe_2_O_3_, indicating that Fe was present predominantly in an oxidized cationic state rather than as metallic Fe. Figure 4e shows the edge position of FeSAC/NC lay between those of FeO (Fe^2+^) and Fe_2_O_3_ (Fe^3+^), suggesting some Fe had the oxidation state of 2+ and others had the oxidation state of 3+. The Fe K‐edge Fourier‐transform extended x‐ray absorption fine structure (FT‐EXAFS) spectra (Figure 4f) showed that the Fe foil and Fe_2_O_3_ exhibited prominent Fe─Fe peaks at radial distances at 2.21 and 2.60 Å, whereas the FeSAC/NC material displayed only a single peak at around 1.63 Å, assignable to Fe─N coordination with no detectable Fe─Fe contribution [43]. The wavelet transform (WT) EXAFS analysis with high resolution in both k and R spaces was carried out for FeSAC/NC, Fe foil and Fe_2_O_3_ (Figure 4g–i). The WT contour plot of FeSAC/NC displayed a single intensity maximum at low R (1.5–2.0 Å) and relatively low k, which was attributed to Fe─N coordination [44]. In contrast, Fe foil and Fe_2_O_3_ exhibited pronounced WT maxima at higher R and k values, originating from Fe─Fe scattering; the absence of any Fe─Fe WT feature in FeSAC/NC indicated that Fe species were present as atomically dispersed iron single atoms rather than Fe or Fe_2_O_3_ clusters [43, 44]. As shown in Figure S18, the Fe K‐edge EXAFS spectrum of the FeSAC/NC material was fitted using an Fe─N single‐scattering path. The simulated curve matched the experimental data, with a low R‐factor of 0.016, indicating a reasonably reliable fit. The best‐fitting parameters gave an Fe─N coordination number of CN = 4.31 ± 0.71 and an average Fe─N bond distance of R = 2.09 ± 0.01 Å. Together with the absence of Fe─Fe coordination in the FT‐EXAFS spectrum, the absence of Fe─Fe WT feature, the fitting results, and the aberration‐corrected HAADF‐STEM observations, the results showed the coordination number was most likely to be Fe─N_4_‐like local coordination environment.

**FIGURE 5:**
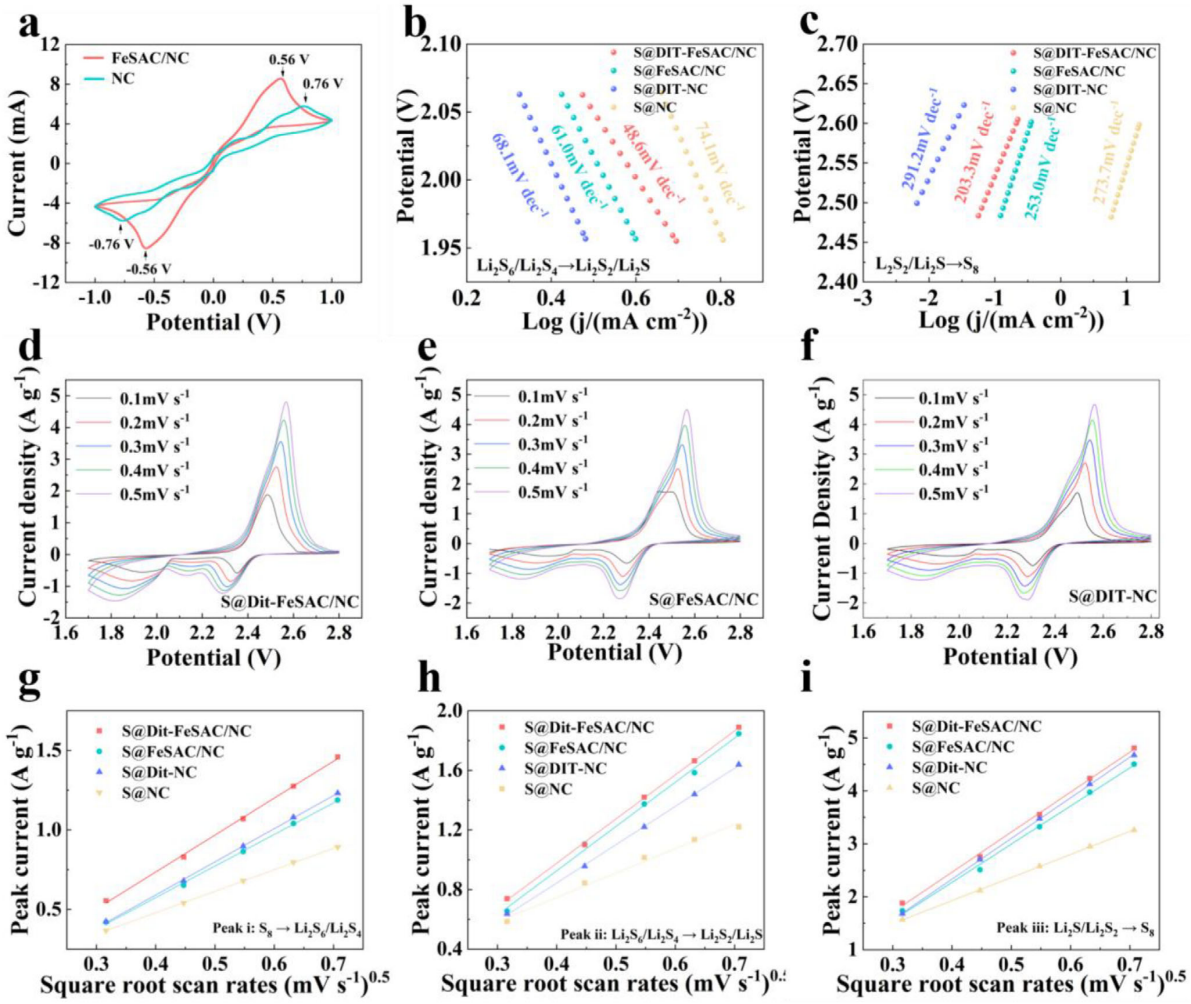
a) CV curves of symmetric cells using the FeSAC/NC and NC electrodes with Li_2_S_6_ electrolyte at a scan rate of 0.5 mV s^−1^. (b) Tafel plots for the reduction process. (c) Tafel plots for the oxidation process. CV curves of the cathodes vs Li metal anode with different scan rates from 0.1 to 0.5 mV s^−1^ for (d) S@DIT‐FeSAC/NC; (e) S@FeSAC/NC; and (f) S@DIT‐NC. The corresponding scan‐rate CV curves of S@NC are shown in Figure S19. Li^+^ ion diffusion properties of S@DIT‐FeSAC/NC, S@FeSAC/NC, S@DIT‐NC and S@NC probed by (g) redox peak i: S_8_ → Li_2_S_6_/Li_2_S_4_; redox peak ii: Li_2_S_6_/Li_2_S_4_ →Li_2_S_2_/Li_2_S; redox peak iii: Li_2_S/Li_2_S_2_→S_8_.

To understand the dynamic electrocatalytic behaviour toward polysulfide conversion, cyclic voltammetry (CV) profiles of symmetric cells for the FeSAC/NC electrodes and NC electrodes in Li_2_S_6_ electrolyte were collected at a scan rate of 0.5 mV s^−1^. Li_2_S_6_ is a soluble intermediate polysulfide, which enables two electrodes to probe both directions of sulfur redox. As depicted in Figure 5a, both cells showed redox peaks; the positive peaks reflected the reduction of Li_2_S_6_ to Li_2_S, and the negative peaks represented the reversible oxidation process. FeSAC/NC exhibited sharper redox peaks and a smaller voltage polarization of 1.12 V as compared to NC (1.52 V), indicative of the kinetically enhanced sulfur redox. To understand the impact of catalytic activity and cathode microstructural design on energy storage performance, four types of S host cathodes were fabricated and assembled into Li─S coin cells for electrochemical testing: (i) S@DIT‐FeSAC/NC (FeSAC/NC made by DIT); (ii) S@ FeSAC/NC (FeSAC/NC made by conventional slurry coating); (iii) S@DIT‐NC (NC without FeSAC made by DIT); (iv) S@NC (NC without FeSAC made by slurry coating). The Tafel slope was derived from the linear scanning voltammetry (LSV) of the reduction process (Figure S19) and oxidation process (Figure S20) for the cells containing Li anodes and the four types of S‐loaded cathodes. Tafel equation: η = a + b×log j, where *η* is the overpotential, *j* is the current density, and *b* is the Tafel slope [45]. A smaller Tafel slope indicates that less overpotential is required to drive the same current density, implying faster charge‐transfer kinetics and more favorable reaction pathways [45]. Tafel slope (Figure 5b,c) analysis revealed that S@DIT‐FeSAC/NC exhibited the smallest slopes in both the reduction (48.6 mV dec^−^^1^) and oxidation (203.3 mV dec^−^^1^) processes, compared to S@FeSAC/NC, S@DIT‐NC, and S@NC. This demonstrated that FeSAC and the vertically aligned structure exhibited faster overall sulfur redox reaction kinetics of sulfur cathodes compared with the vertically aligned structure without the catalytic sites and the randomly mixed structure without the catalytic sites. CV curves of the four types of cathodes against a Li anode were recorded under a voltage range of 1.7–2.8 V at scan rates ranging from 0.1 to 0.5 mV s^−^^1^ (Figure 5d–f and Figure S21). The cathodic peak at approximately 2.3 V was assigned to the reduction of elemental sulfur to soluble long‐chain polysulfides, while the cathodic peak at approximately 1.8–2.0 V corresponded to the subsequent conversion of soluble polysulfides into insoluble Li_2_S_2_/Li_2_S. During the anodic scan, the oxidation response centered at approximately 2.5 V was associated with the oxidation of Li_2_S/Li_2_S_2_ to soluble polysulfides and ultimately to elemental sulfur. The higher current response of the first reduction peak at 2.3 V than the second reduction peak at 1.8–2.0 V indicated faster sulfur redox kinetics for the reduction of elemental sulfur to soluble long‐chain polysulfides compared with the more sluggish solid‐state precipitation and further reduction of polysulfides into insoluble short‐chain products (Li_2_S_2_/Li_2_S). Nevertheless, S@DIT‐FeSAC/NC exhibited the highest current response at 1.8–2.0 V among the four types of cathodes, indicating improved sulfur redox kinetics for both reduction reactions with FeSAC and the DIT structure. The linear relationship between the peak current (I_p_) and the square root of the scan rate (v^0.5^) (Figure 5g–i) was analyzed for qualitative comparison amongst the different types of electrodes [46]. The S@DIT‐FeSAC/NC electrode exhibited the highest slopes for all redox peaks amongst the S@FeSAC/NC, S@DIT‐NC and S@NC electrodes, indicating faster Li^+^ transport and sulfur redox kinetics. To further investigate the effect of the DIT architecture on Li^+^ transport kinetics, Galvanostatic intermittent titration technique (GITT) measurements were conducted (Figure S22). The S@DIT‐FeSAC/NC electrode exhibited lower voltage polarization throughout the test than S@FeSAC/NC, S@DIT‐NC, and S@NC cathodes. The calculated $D_{Li^{+}}$ values (Figure S23) further confirmed that S@DIT‐FeSAC/NC delivered the highest and most stable Li‐ion diffusion coefficient, followed by S@FeSAC/NC, S@DIT‐NC and S@NC, indicating its improved ion transport kinetics. This result was consistent with the pore tortuosity analysis estimated from the cross‐sectional SEM images of the electrodes shown earlier, where the DIT electrode exhibited a substantially reduced tortuosity (τ = 3.68) compared with the conventional electrode (τ = 14.4), providing more direct ion transport pathways and reducing polarization. To further evaluate the liquid‐to‐solid sulfur conversion kinetics, Li_2_S nucleation tests were performed by chronoamperometry at 2.05 V (Figure S24). Compared with S@DIT‐NC and S@NC, the Fe‐containing electrodes exhibited earlier and more pronounced nucleation current peaks, indicating accelerated Li_2_S nucleation kinetics induced by the Fe─N_4_ active sites. Notably, the S@DIT‐FeSAC/NC electrode delivered the highest nucleation current and the largest Li_2_S precipitation capacity (108.2 mAh g^−^^1^), demonstrating the synergistic effect of the directionally aligned porous architecture and the Fe─N_4_ catalytic sites in promoting Li_2_S formation [47].

**FIGURE 6:**
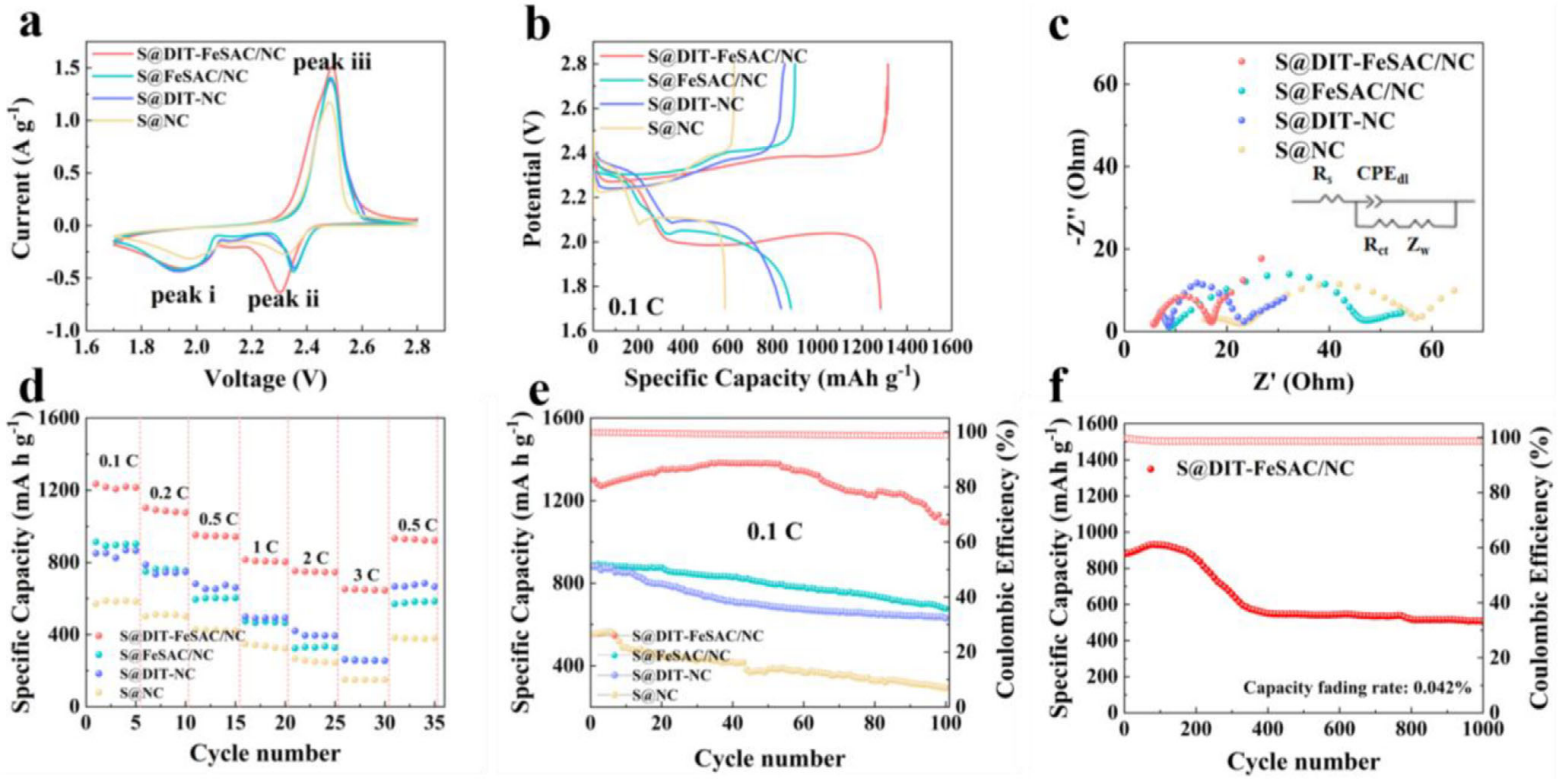
Electrochemical testing of the S@DIT‐FeSAC/NC, S@FeSAC/NC, S@DIT‐NC and S@NC cathodes in Li─S battery cells for (a) CV curves at 0.05 mV s^−1^. (b) GCD profiles at 0.1 C. (c) Nyquist plot. (d) Rate capability performance. (e) Cycling performance at 0.1 C for 100 cycles. (f) Long cycling performance of the S@DIT‐FeSAC/NC cathode at 0.5 C for 1000 cycles.

Figure 6a compares the CV curves of the four types of cathodes under a voltage range of 1.7–2.8 V at a scan rate of 0.05 mV s^−1^. Compared with the control cathodes, the S@DIT‐FeSAC/NC electrode exhibited the highest current responses, indicating faster sulfur redox kinetics. Figure 6b displays the galvanostatic charge‐discharge (GCD) curves of the four types of cells at a rate of 0.1 C. The S@DIT‐FeSAC/NC cathode achieved a discharge capacity of 1299.2 mAh g^−^^1^, surpassing that of S@FeSAC/NC (823.5 mAh g^−^^1^), S@DIT‐NC (838.7 mAh g^−1^), and S@NC (587.1 mAh g^−^^1^), and demonstrated a longer charge‐discharge plateau. Furthermore, S@DIT‐FeSAC/NC exhibited a smaller voltage gap of 0.34 V compared to 0.38 V for S@FeSAC/NC, 0.37 V for S@DIT‐NC, and 0.40 V for S@NC, indicating that the electrocatalyst and rational cathode microstructural design reduced cell polarization and promoted efficient electron and ion transport. For consistent comparison, 2.10 V was used as a common operational boundary to separate the high‐ and low‐voltage discharge capacities for all electrodes [48]. The S@DIT‐FeSAC/NC electrode exhibited a higher. *Q_L_*/*Q_H_* ratio (3.76) than S@FeSAC/NC (2.15), S@DIT‐NC (2.10) and S@NC (1.97), indicating a larger capacity contribution from the low‐voltage sulfur conversion region. Electrochemical impedance spectroscopy (EIS) was employed to further investigate the reaction kinetics of the four types of cells (Figure 6c) [49]. The diameter of the first semicircle of the Nyquist plot indicates the charge transfer resistance of the electrodes [50]. The results clearly showed that S@DIT‐FeSAC/NC had a lower charge transfer resistance than the other types of cathodes due to the vertically aligned pore arrays, which facilitated Li^+^ ion transport. A range of charge and discharge rates, from 0.1 C to 3 C, was applied to the four types of cells, followed by returning to 0.5 C. Figure 6d demonstrates that the S@DIT‐FeSAC/NC cell maintained the highest capacity across the different charge and discharge rates, and retained a high capacity of 933.5 mAh g^−^^1^ even after returning to 0.5 C. The cycling performance of the four types of cells was evaluated at 0.1 C (Figure 6e), the S@DIT‐FeSAC/NC cell delivered a discharge capacity of 1299.2 mAh g^−^^1^ in the first cycle. The capacity of S@DIT‐FeSAC/NC then stabilized between 1300 and 1400 mAh g^−1^ till 50 cycles, and ∼1093.9 mAh g^−1^ at 100 cycles, which was higher than those of all the other comparison cathodes of S@FeSAC/NC, S@DIT/NC, and S@NC. The slight increase in capacity of S@DIT‐FeSAC/NC during the initial cycles was attributed to a gradual electrochemical activation process. During repeated cycling, progressive activation of the sulfur‐containing electrochemically active interfaces may improve the accessibility and utilization of sulfur species, allowing initially less‐accessible active material to participate more effectively in the reversible sulfur redox reactions. Similar increases in discharge capacity during the initial cycling stage have been reported in Li─S batteries and were associated with gradual electrochemical activation [51, 52]. In S@DIT‐FeSAC/NC, the interconnected porous architecture provided accessible reaction interfaces and ion‐transport pathways, while the Fe─N_4_ single‐atom sites promoted sulfur redox conversion, which may collectively facilitate the gradual activation and utilization of sulfur species. After reaching the maximum capacity, the capacity gradually decreased upon prolonged cycling. The results of electrode structural tortuosity analysis and electrochemical testing show that despite the higher electrode thickness of the DIT electrodes than the randomly structured electrodes, the vertically aligned microstructure of the DIT electrodes facilitated electrolyte uptake, reduced polarization, improved ion transport, and increased sulfur utilization under the same sulfur mass loading and electrolyte volume. To further demonstrate the advantages of the 3D‐oriented structure under high‐loading conditions, an S@DIT‐FeSAC/NC cathode with an S mass loading of 5 mg cm^−^^2^ was prepared, which represented a practically relevant high sulfur loading commonly investigated in high‐loading Li─S batteries [53, 54, 55]. The cell delivered a high areal capacity of 5.6 mAh cm^−^^2^ (1120 mAh g^−1^) at 0.1 C (Figure S25), showing that the anisotropic cathode structure enabled efficient active material utilization with large access surface area and fast ion diffusion deep in the cathode even at high mass loading. Finally, the long‐term cycling performance of S@DIT‐FeSAC/NC was assessed at 0.5 C (Figure 6f), the cell exhibited an initial capacity of 878.8 mAh g^−^^1^ and maintained a capacity of 505.9 mAh g^−^^1^ after 1000 cycles, with an average capacity decay rate of only 0.042% per cycle. Representative galvanostatic discharge–charge profiles at the first, 100^th^, 500^th^, and 1000^th^ cycles are shown in Figure S26. A slight increase in capacity was observed during the initial cycles, which may be attributed to the gradual activation of the porous cathode. With prolonged cycling, the characteristic voltage plateaus were maintained. To investigate the suppression of the polysulfide shuttle effect during cycling, post‐cycling anode characterization was performed where the morphology of the Li metal anodes after 100 cycles at 0.1 C was characterized by SEM. As shown in Figure S27, the Li anode paired with the S@NC cathode exhibited abundant irregular deposits and severe surface roughening, indicating extensive side reactions induced by dissolved polysulfides. The S@FeSAC/NC and S@DIT‐NC cathodes alleviated the surface contamination to different extents, and the S@DIT‐FeSAC/NC cathode exhibited the smoothest surface with the least irregular deposits, reflecting the combined beneficial effects of catalytic sites and directional architecture. Notably, the Li anode cycled with the S@DIT‐FeSAC/NC cathode maintained the smoothest surface with substantially fewer by‐products, suggesting that the synergistic combination of the Fe─N_4_ catalytic sites and the DIT architecture effectively suppressed polysulfide shuttling and mitigated parasitic reactions during cycling. The reproducibility of the electrochemical performance was further verified by repeat measurements of the cycling performance and rate capability, which showed consistent overall trends among the different cathodes (Figures S28 and S29). The capacity and cycling stability of S@DIT‐FeSAC/NC were compared with those of cathodes using various single‐atom catalysts in Li─S batteries (e.g. Ni [56], Co [57], etc.) (comparison summarized in Table S1). Sulfur loading (active material) and electrolyte volume were kept the same amongst the different types of electrodes for a fair comparison of gravimetric capacity and active material utilization, and to show the effects of electrode structure and FeSAC. The DIT architecture resulted in a thicker electrode than the conventional slurry‐coated electrode. Although this porous architecture improved electrolyte infiltration, Li^+^ transport, and accommodated volume changes of electrodes induced by sulfur conversion in Li─S batteries, future work includes optimizing electrode structure to further improve reaction kinetics and cycling stability, applying calendering that has been studied in Li ion batteries to Li─S batteries to reduce thickness and increase electrode density and volumetric energy density, and studying performance at different S loadings.

## 3 Conclusions

We fabricated Fe─N_4_ single‐atom catalysts dispersed on C_3_N_4_ (FeSAC/NC) and self‐assembled into a vertically aligned electrode structure by directional ice templating (DIT) for the S host cathode of Li─S batteries. DFT calculations show enhanced adsorption strength, enhanced electronic states near the Fermi level, and reduced reaction energy change for Li_2_S_2_‐to‐Li_2_S liquid‐solid bidirectional S transformation on the Fe─N_4_ sites. Aberration‐corrected HAADF‐STEM and x‐ray absorption spectroscopy support the presence of atomically dispersed Fe species with a Fe─N_4_‐like local coordination environment on the g‐C_3_N_4_ support. A scalable DIT fabrication instrument is built to make S@DIT‐FeSAC/NC, where the material self‐assembles into a vertically aligned structure that maximizes the exposure of Fe─N_4_ active sites, speeds up S redox reactions at the atomic level, and Li^+^ ion diffusion at the microscale. Benefiting from the multi‐length scale design, Tafel plots show that S@DIT‐FeSAC/NC accelerates the redox kinetics and reversibility of the cathode. The Li─S coin cells containing the S@DIT‐FeSAC/NC cathode exhibit the highest discharge capacity of 1299.2 mAh g^−1^ at 0.1 C, the highest rate capability as the charge/discharge rate increases, the lowest impedance, and long cycle stability, retaining 505.9 mAh g^−1^ after 1000 cycles at 0.5 C with a low average capacity fade of 0.042% per cycle. At a sulfur loading of 5.0 mg cm^−^^2^, a high areal capacity of 5.6 mAh cm^−^^2^ (1120 mAh g^−1^) at 0.1 C is achieved, indicating efficient utilization of the Fe─N_4_ active sites even at high mass loading. Beyond Li─S batteries, the novel integration of single‐atom catalyst sites in vertically oriented DIT structures holds potential for enhancing the kinetics of redox reactions and rates of ion transport for a range of electrocatalytic and energy storage applications.

## 4 Methods

### 4.1 DFT Calculations

Density Functional Theory (DFT) calculations were performed using the CP2K/Quickstep package [58, 59, 60]. Structural optimizations were carried out within the Born–Oppenheimer framework. The Perdew–Burke–Ernzerhof (PBE) functional [61] within the generalized gradient approximation (GGA) was employed to describe exchange–correlation interactions. A mixed Gaussian and plane‐wave (GPW) approach was adopted, using a plane‐wave cutoff energy of 500 Ry together with a double‐ζ valence basis set with polarization functions (DZVP‐MOLOPT‐SR‐GTH) [62].

The computational cell had dimensions of 28.54 Å × 28.54 Å × 22 Å, with periodic boundary conditions applied in the x and y directions and the z direction treated as nonperiodic, corresponding to a two‐dimensional periodic model. Electrostatics were treated using the Martyna–Tuckerman Poisson solver (PSOLVER MT), with PERIODIC XY specified in both the CELL and POISSON sections. This setup avoids artificial interactions between periodic images and eliminates possible electrostatic coupling arising from dipole moments along the z direction [63]. Accordingly, the z dimension represents the finite nonperiodic simulation domain rather than a vacuum separation between periodically repeated slabs. For the entire system, the approximately 22 Å cell dimension along the nonperiodic z direction was considered sufficient to satisfy the cell‐size requirement of the MT treatment [64, 65, 66], which requires the cell length along a nonperiodic direction to be sufficiently large relative to the spatial extent of the complete charge distribution. To further verify this choice, additional calculations were performed with the z dimension increased by 50% to 33 Å, which resulted in an almost negligible change in the calculated adsorption energy (0.00016 eV). Owing to the large lateral dimensions of the simulation cell, Brillouin‐zone sampling was restricted to the Γ‐point.

Electronic self‐consistent field (SCF) calculations were converged to a threshold of 5.0 × 10^−^^6^ a.u. Geometry optimizations were performed using the Broyden–Fletcher–Goldfarb–Shanno (BFGS) algorithm and were considered converged when the maximum atomic force was below 4.5 × 10^−^^4^ Ha Bohr^−^^1^.

The minimum energy path (MEP) was determined using the climbing‐image nudged elastic band (CI‐NEB) method as implemented in the CP2K BAND framework. The reaction pathway was discretized into five images generated by interpolation between the optimized initial‐state (IS) and final‐state (FS) structures. Spin polarization was explicitly considered for Fe‐SAC‐C_3_N_4_ within the PBE‐GGA DFT framework. The calculation converged to equal α‐ and β‐electron populations (520 electrons each), with an integrated absolute spin density of 4.239 × 10^−^^4^ e, indicating an essentially spin‐compensated electronic state within the present computational framework. This result should be interpreted within the limitations of the employed exchange–correlation treatment and does not exclude the possibility of localized Fe magnetic moments under alternative electronic‐structure treatments. For Li_2_S adsorption on FeSAC/NC, the optimized structure exhibits an Fe─S distance of 2.068 Å, indicating direct interaction between the adsorbed Li_2_S species and the Fe─N_4_ site. Hirshfeld population analysis gives a net charge of approximately −0.202 e on the adsorbed Li_2_S fragment, indicating adsorption‐induced charge redistribution and interfacial polarization associated with the Fe─S interaction.

### 4.2 Materials

Iron(III) chloride hexahydrate (ACS reagent, 97%, Merck Life Sciences); dicyandiamide (99.0%, Merck Life Sciences); ammonium chloride (ACS reagent, 99.5%, Merck Life Sciences); lithium sulfide (Merck Life Sciences); sulfur  (reagent grade, purified by sublimation, 100 mesh particle size, Merck Life Sciences); 1,3 dioxolane (DOL, Merck Life Sciences), 1,2‐dimethoxyethane (DME, Merck Life Sciences. Battery components, including conductive C65, polypropylene separator (Celgard 2500), coin cells (CR2032), lithium metal disks, aluminum foil, and electrolyte (0.5 M LiTFSI + 0.5 M LiNO_3_ in DOL/DME (vol 50:50)) were purchased from Xiamen TOB New Energy Technology Co., LTD.

### 4.3 Preparation of Fe‐SAC/NC and NC

First, 1 g dicyandiamide (C_2_H_4_N_4_) was mixed into 30 mL DI water with continuous stirring at 50°C for 2 h to form a homogeneous solution. Then 150 mg Ferric chloride trihydrate (FeCl_3_·6H_2_O) and 3 g ammonium chloride NH_4_Cl were added to the above solution, and the mixture was stirred at 50°C for 2 h. Accordingly, the solution was subject to a freeze‐drying process in vacuum for 12 h to produce a yellow powder. Subsequently, the FeSAC/NC was annealed at 550°C for 2 h with a heating rate of 5°C min^−1^ under an argon atmosphere. The obtained powder was added to the 20% H_2_SO_4_ solution, which was stirred for 24 h to remove the metal clusters. Next, the obtained yellow powder was collected by centrifugation and washed three times with DI water. Finally, the FeSAC/NC was obtained by vacuum drying for 24 h. Similarly, NC was prepared without FeCl_3_·6H_2_O.

### 4.4 Preparation of S@DIT‐FeSAC/NC, S@FeSAC/NC, S@DIT‐NC and S@NC

Sulfur composites were prepared by the melt‐diffusion method. Sulfur and the host material (FeSAC/NC or NC) were mixed at a ratio of 4:1 which were grounded in a mortar for 30 min. Then the mixture was put into a glass bottle and heated at 155°C for 12 h to enable the diffusion of the sulfur substance into the host materials. The S@DIT‐FeSAC/NC and S@DIT‐NC electrodes were prepared by DIT through freezing on a cold peltier after slurry coating and were subjected to a freeze‐drying process in vacuum for 24 h. The S@FeSAC/NC and S@NC electrodes were fabricated by slurry coating and conventional drying through heating. All types of electrodes consist of S+host material, C65, and a binder (50 wt.% carboxymethyl cellulose + 50 wt.% styrene‐butadiene rubber) with a mass ratio of 8:1:1. We controlled the sulfur loading (2 mg/cm^2^ ± 2%) to be the same for both the slurry‐cast and DIT electrodes. Due to vertically directional structural growth and volume expansion during the DIT process, the thickness of the DIT electrodes was ∼230 µm, which is much larger than that of the slurry‐coated electrodes (∼100 µm).

### 4.5 Characterization

Structural and morphological observations of the single atom were studied by Aberration‐corrected Transmission Electron Microscopy (AC‐TEM, JEOL ARM300CF) with a beam energy of 80 keV and a 40 mm condenser lens aperture (32 mrad), an ADF detector (inner angle 47 mrad), and a beam current of ≈46 pA, and Transmission Electron Microscopy (TEM, JEOL 2100F). Morphological observations of the directional ice templates were characterized by Scanning Electron Microscopy (SEM, Zeiss Auriga Cross Beam FIB‐SEM). For post‐cycling characterization, the coin cells were disassembled after 100 cycles at 0.1 C, and the Li metal anodes were carefully collected and rinsed with DME to remove residual electrolyte and soluble species. After drying, the surface morphology of the recovered Li anodes was examined by SEM (TFS Helios Hydra Dual Beam Plasma FIB‐SEM). For pore size distribution measurement, individual pores were identified as connected components within the binary pore mask. The area of each labeled pore was measured in pixels and converted to physical units using the calibrated pixel size. An equivalent circular diameter was calculated based on the measured pore area, and the resulting diameters were used to generate probability‐normalized histograms for pore size distribution analysis. Fe loading of FeSAC/NC powder was analyzed by ICP‐OES using an Optima 8000, PerkinElmer. Sample preparation for ICP‐OES follows standard procedures (e.g [67]), where the samples were calcined in air at 800°C for 6 h to remove the carbonaceous matrix and other impurities. The resulting residue was subsequently digested in freshly prepared solution (HNO_3_:HCl = 3:1, v/v) for 6 h to dissolve the Fe‐containing species. The obtained solution was then diluted for ICP analysis. Pore tortuosity τ was estimated by comparing the Li ion effective diffusive flux through the measured pore network (F_p_) with the flux through an open volume of the same size (F_cv_) using

(1) $$\mathrm{Fp}\;=-\mathrm{AD}\frac{\varepsilon \;}{\tau }\frac{\Delta C}{L}$$

and

(2) $$\mathrm{Fcv}\;=-\mathrm{AD}\frac{\Delta C}{L}$$

where ε is the pore volume, D is the diffusivity of Li ions in the electrolyte, and ΔC is the imposed, starting Li ion concentration difference across the sub‐domain of thickness L and cross‐sectional area A [28]. Calculating the ratio of Equations (1) and (2) yields τ  =  Fcv/Fp. The crystalline phases of the prepared materials were characterized by x‐ray diffractometer (XRD, Bruker D2 Phaser) equipped with Cu Kα radiation (λ = 1.5406 Å). The surface valence states and chemical environment were characterized by x‐ray photoelectron spectroscopy (XPS, Thermo Fisher K‐Alpha+). The Fe K‐edge x‐ray absorption fine structure (XAFS) spectra of all samples were collected at Diamond Light Source on beamline B18 in fluorescence mode. The incident x‐ray beam was monochromated using a Si(111) crystal set. A Pt‐coated optical branch was employed with the harmonic rejection mirror inserted, and a 140 µm pyrolytic carbon filter was used in the white‐beam path. Fe K‐edge fluorescence spectra were collected in continuous‐scanning mode over the energy range of 6912–7962 eV, with an average energy spacing of approximately 0.30 eV per point. The fluorescence signal was collected in a near‐90° geometry to minimize the elastic scattering background. Energy calibration was performed using a simultaneously measured Fe foil reference, and the absorption edge position of the reference spectrum was calibrated to the known theoretical value. For each sample, four consecutive scans were collected, aligned, and averaged to improve the signal‐to‐noise ratio and obtain more reliable spectra. The raw fluorescence data were normalized to the incident intensity I_0_, and a self‐absorption correction was applied. XANES/EXAFS data were processed using the Athena module of the Demeter package (version 0.9.26) for energy calibration, background subtraction, and normalization, with E_0_ defined as the energy corresponding to the maximum in the first derivative of the absorption edge. The normalized m(E) spectra were used for XANES analysis, while the EXAFS oscillations were converted into k space, weighted by k^3^, and Fourier transformed into R space. Subsequent EXAFS fitting was performed using Artemis (version 0.9.26) over the ranges of k = 3–13 Å^−1^ and R = 1.1–2.5 Å. Nitrogen adsorption/desorption isotherms were obtained at a temperature of 77 K on the Autosorb iQ Quantachrome 6100. The Brunauer–Emmett–Teller (BET) method was employed to calculate the specific surface area. A linear fit was performed in the relative pressure range of P/P_0_ = 0.05 – 0.30. Typically, 5 points within this region were selected for fitting. The BET plot was constructed by plotting P/P_0_ versus 1/[V(P_0_/P−1)], yielding a linear relationship. From the slope (k) and intercept (i) of the fitted line, the monolayer adsorption capacity (Vm) was calculated according to: $K=\frac{C-1}{VmC}$, $i=\frac{1}{VmC}$, $Vm=\frac{1}{k+i}$. The specific surface area (S_bet_) was then determined using: $S_{BET}=\frac{Vm\mathrm{Na}Am}{Mm*m}$. For nitrogen adsorption, this equation can be simplified to: *S_BET_* =  4.35 × *V_m_*. Thermogravimetric analysis (TGA) was performed using a NETZSCH Jupiter STA 449 C instrument. The samples were heated from room temperature to 500°C at a heating rate of 5°C min^−^^1^.

### 4.6 Electrochemical Testing

Standard CR2032 coin cells with a Celgard 2400 membrane as the separator were assembled in a glovebox filled with argon for electrochemical testing. For the symmetrical cells, the FeSAC/NC and NC electrodes were prepared by dissolving 10 mg FeSAC/NC and NC in 5 mL ethanol and dropping them evenly on the carbon paper (CP). A Li_2_S_6_ solution (0.2 mol L^−1^) was prepared by mixing Li_2_S and S with a molar ratio of 1:5 in the 1,2‐dimethoxyethane (DME)/1,3‐dioxolane (DOL) solution containing lithium bis(trifluoromethanesulfonyl)imide (LiTFSI, 0.5 M) and LiNO_3_ (0.5 M) and used as the electrolyte (40 µL). CV tests of the symmetric cells were performed at a scan rate of 0.5 mV s^−1,^ with the voltage ranging from −1.0 to 1.0 V. For full cells, the electrolyte contained 0.5 M LiTFSI in a solvent mixture of DOL and DME (1:1 in volume) with 0.5 M LiNO_3_. For the comparative electrochemical measurements at a sulfur loading of 2 mg cm^−^^2^, a fixed electrolyte volume of 30 µL was used for all the full cells in coin cells. The electrolyte‐to‐sulfur (E/S) ratio used in the coin cells with typical sulfur loading was 7.16 µL mg^−1^, similar to 8 µL mg^−1^ [68] and 7.5 µL mg^−1^ [69] in the literature, to ensure the performance difference was due to the cathode material and electrode structure rather than any potential electrolyte starvation that could obscure the intrinsic effects of the electrode design. Future work includes longer cycling testing in lean‐electrolyte conditions (e.g., E/S ratio <5 µL mg^−1^). Commercial lithium metal foil (16 mm in diameter and 250 µm in thickness) was used as the anode. The negative‐to‐positive electrode capacity ratio (N/P ratio) was calculated based on the theoretical capacities of Li (3860 mAh g^−^^1^) and S (1672 mAh g^−^^1^). The calculated N/P ratios were approximately 15.4 and 6.2 for sulfur loadings of 2 and 5 mg cm^−^^2^, respectively. LSV was carried out, and CV measurements at varied scan rates were carried out on the Biologic electrochemical workstation. GCD tests and rate/cycling performance were conducted on an Arbin battery testing system with a voltage range of 1.7–2.8 V (vs. Li/Li^+^). Galvanostatic intermittent titration technique (GITT) measurements were conducted to evaluate the Li^+^ diffusion kinetics of the electrodes. Li─S coin cells were assembled using S@DIT‐FeSAC/NC, S@FeSAC/NC, S@DIT‐NC, and S@NC as the cathode and Li metal as the anode. The cells were first charged to 2.8 V and then discharged by applying a constant current pulse corresponding to 0.1 C for 600 s, followed by an open‐circuit relaxation period of 1200 s to allow the cell potential to approach equilibrium. This pulse–relaxation sequence was repeated throughout the discharge process within the voltage range of 2.8‐1.7 V. The Li^+^ diffusion coefficient (DLi^+^) was calculated from the GITT profiles according to: DLi^+^​ = $\frac{4}{\pi \tau }$ ​ ($\frac{mBVm}{\mathrm{MBA}}$​)^2^($\frac{\Delta Es}{\Delta Et}$)^2^. where τ is the duration of the current pulse, mB is the mass of active sulfur, VM is the molar volume of sulfur, MB is the molar mass of sulfur, A is the geometric area of the electrode, ΔEt is the transient voltage change during the current pulse (excluding the ohmic drop), and ΔEs is the steady‐state voltage change between the equilibrium voltages before and after each current pulse, obtained during the relaxation period. For Li_2_S nucleation experiments, Li─S coin cells were assembled using the S@DIT‐FeSAC/NC, S@FeSAC/NC, S@DIT‐NC, and S@NC as the cathode and Li metal as the anode. A 0.2 M Li_2_S_8_ electrolyte was prepared by dissolving Li_2_S and S in a molar ratio of 1:7 in DOL/DME (1:1, vol) containing 0.5 M LiTFSI and 0.5 M LiNO_3_. The cells were first discharged galvanostatically to 2.05 V and then held at 2.05 V to record the current–time response associated with Li_2_S nucleation and growth. The integrated Li_2_S precipitation capacity was calculated by integrating the positive current response over time and dividing by sulfur mass. All specific capacities reported in this work are normalized to the mass of sulfur.

### 4.7 Statistical Analysis

Electrochemical capacities, current densities, and related kinetic parameters were normalized to the sulfur mass in the corresponding cathodes unless otherwise stated. No mathematical transformation or outlier exclusion was applied to the experimental data. Electrochemical reproducibility was evaluated using independently assembled cells tested under identical conditions. The reproducibility of the 0.1 C cycling performance and rate capability was evaluated using n = 5 and n = 3 independently for assembled cells, respectively. Representative repeat measurements are provided in the Supporting Information. The Fe content determined by ICP‐OES is presented as the mean ± standard deviation (SD) of n = 3 independently prepared samples, yielding Fe content of 1.08 ± 0.09 wt.%. Individual electrochemical curves are presented unless otherwise specified. No inferential statistical tests were performed.

## Author Contributions

**Lin Shen**: methodology, data curation, investigation, formal analysis, writing – original draft. **Yijia Zou**: methodology, data curation, investigation, writing – original draft, formal analysis. **Yuming Wang**: data curation. **Erya Gao**: data curation. **Christopher S. Allen**: data curation, writing – review and editing. **Tianyi Zhang**: data curation. **Stephen J. Skinner**: writing – review and editing, supervision. **Nigel P. Brandon**: supervision, writing – review and editing. **Chun Huang**: conceptualization, methodology, supervision, funding acquisition, writing – review and editing, investigation, resources.

## Conflicts of Interest

The authors declare no conflicts of interest.

## Supporting information




**Supporting File**: advs77768‐sup‐0001‐SuppMat.pdf.

## Data Availability

The data that support the findings of this study are available in the supplementary material of this article.
