# Supplementary material for: Synergistic Integration of Fe–N4 Single‐Atom Sites and Directionally Aligned Electrode Architecture for High‐Performance Lithium–Sulfur Batteries
Source: Adv Sci (Weinh). 2026 Sep 11:e77768. Online ahead of print. doi: 10.1002/advs.77768 (PMC13564186; doi:10.1002/advs.77768)
Supplement: Supplementary file 1 [file ADVS-9999-e77768-s001.pdf]

Supporting Information

Synergistic Integration of Fe–N_4 Single-Atom Sites and Directionally Aligned Electrode Architecture for High-Performance Lithium–Sulfur Batteries

*Lin Shen[1], Yijia Zou[1], Yuming Wang[1], Erya Gao[1], Christopher S. Allen[2], Tianyi Zhang[1] Stephen J. Skinner[1], Nigel P. Brandon[3], and Chun Huang[1,4,5]**

[1] Department of Materials, Imperial College London, Exhibition Road, London SW7 2AZ, UK

[2] Electron Physical Science Imaging Centre (ePSIC), Diamond Light Source, Didcot OX11 0DE, UK

[3] Department of Earth Science and Engineering, Imperial College London, Exhibition Road,London SW7 2AZ, UK

[4] The Faraday Institution, Didcot OX11 0RA, UK

[5] Research Complex at Harwell, Harwell Campus, Didcot OX11 0FA, UK

Corresponding author's email address: a.huang@imperial.ac.uk

Keywords: lithium-sulfur batteries; directional ice templating; single-atom catalysts; sulfur redox kinetics; multi-length scale structure

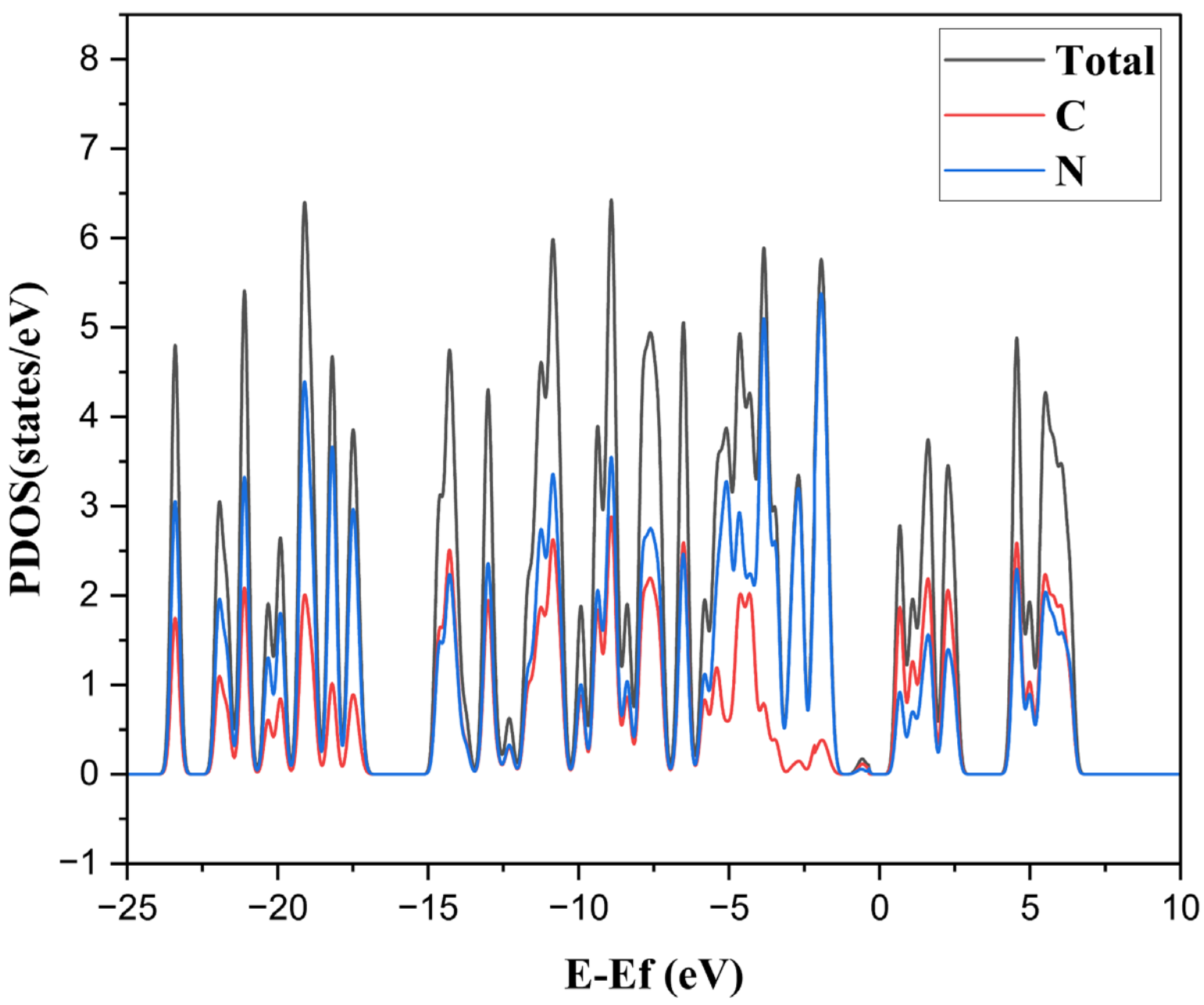

Figure S1. The Partial Density of States (PDOS) of NC.

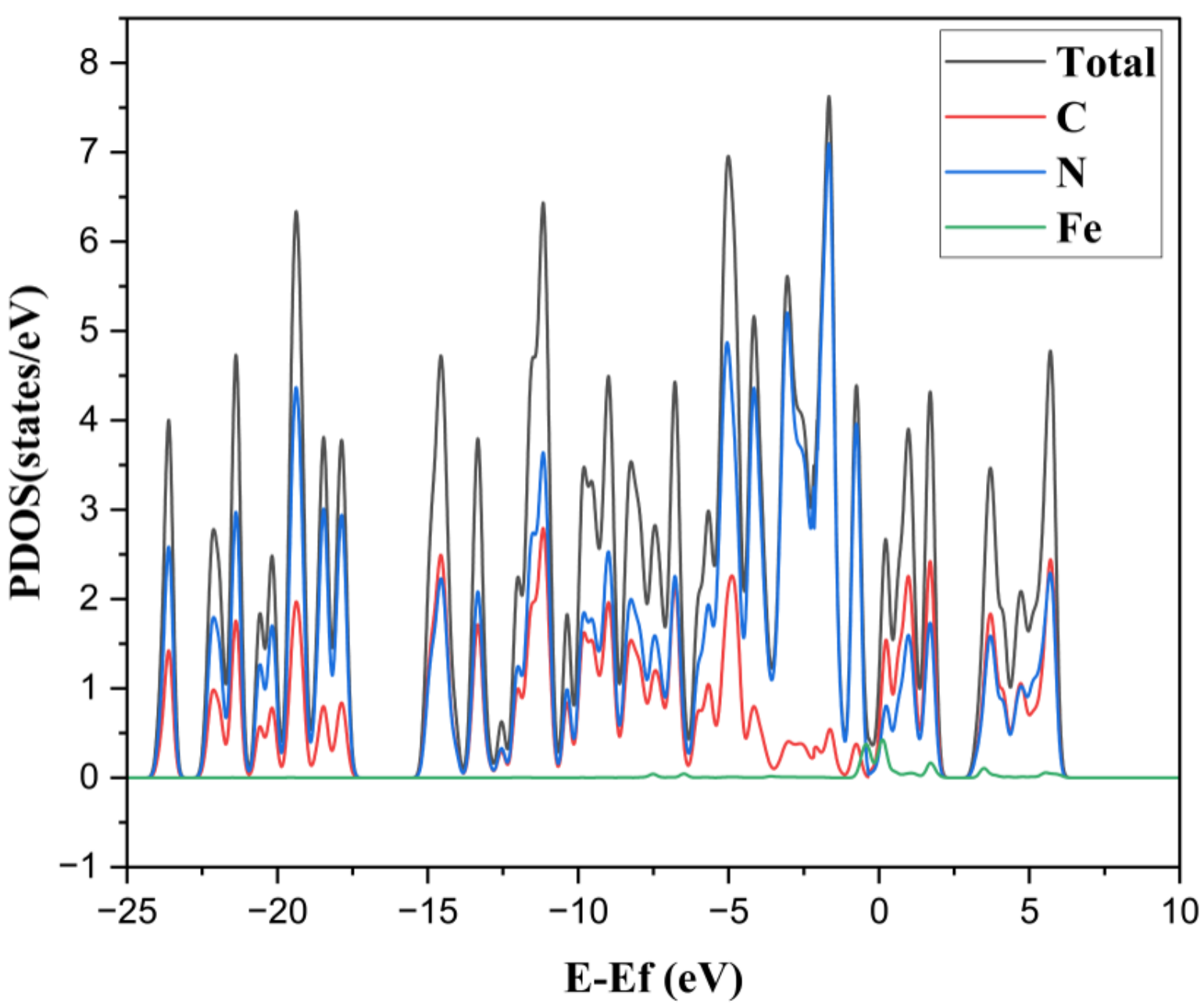

Figure S2. The Partial Density of States (PDOS) of FeSAC/NC.

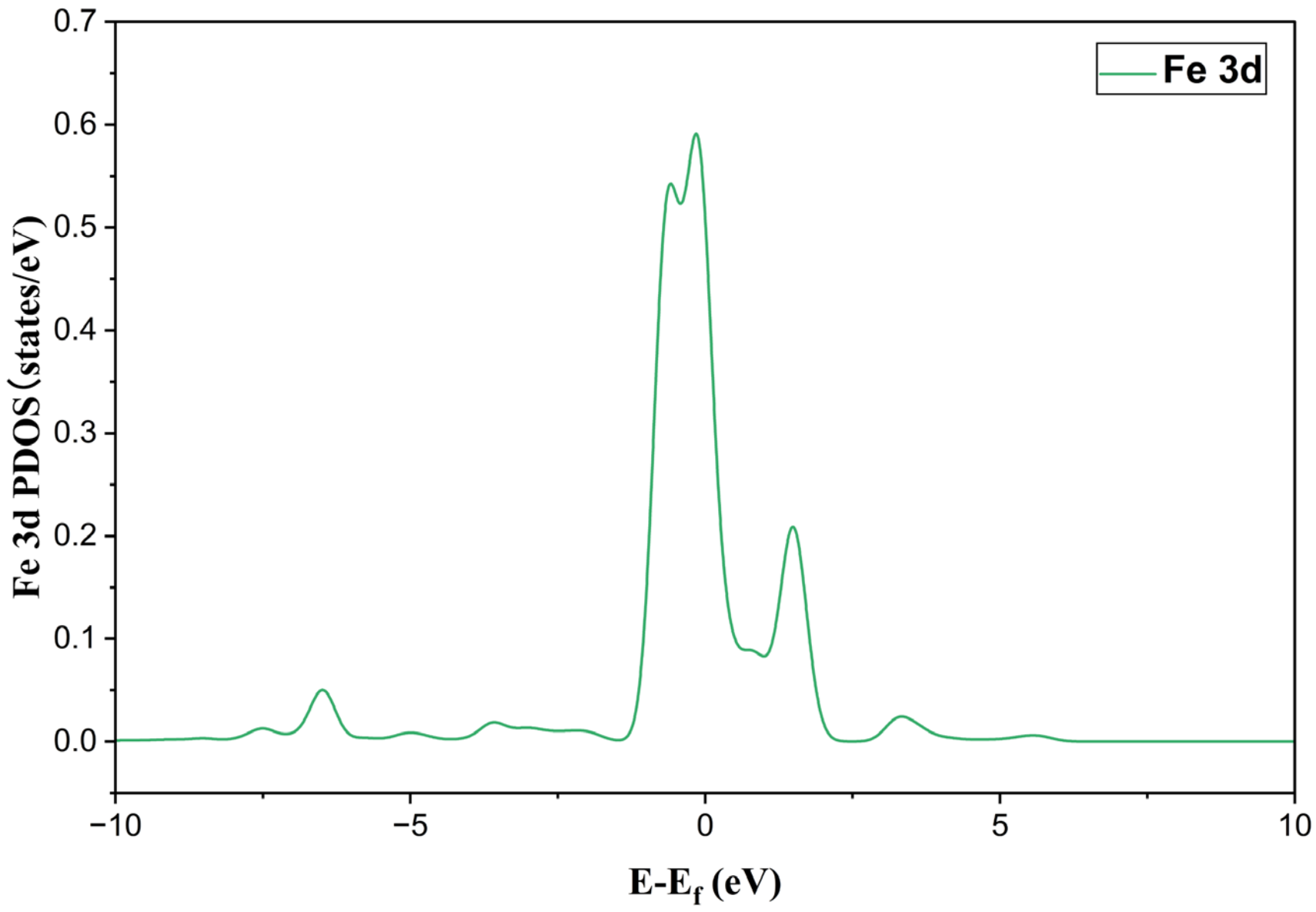

Figure S3. PDOS of the Fe 3d-derived states in FeSAC/NC.

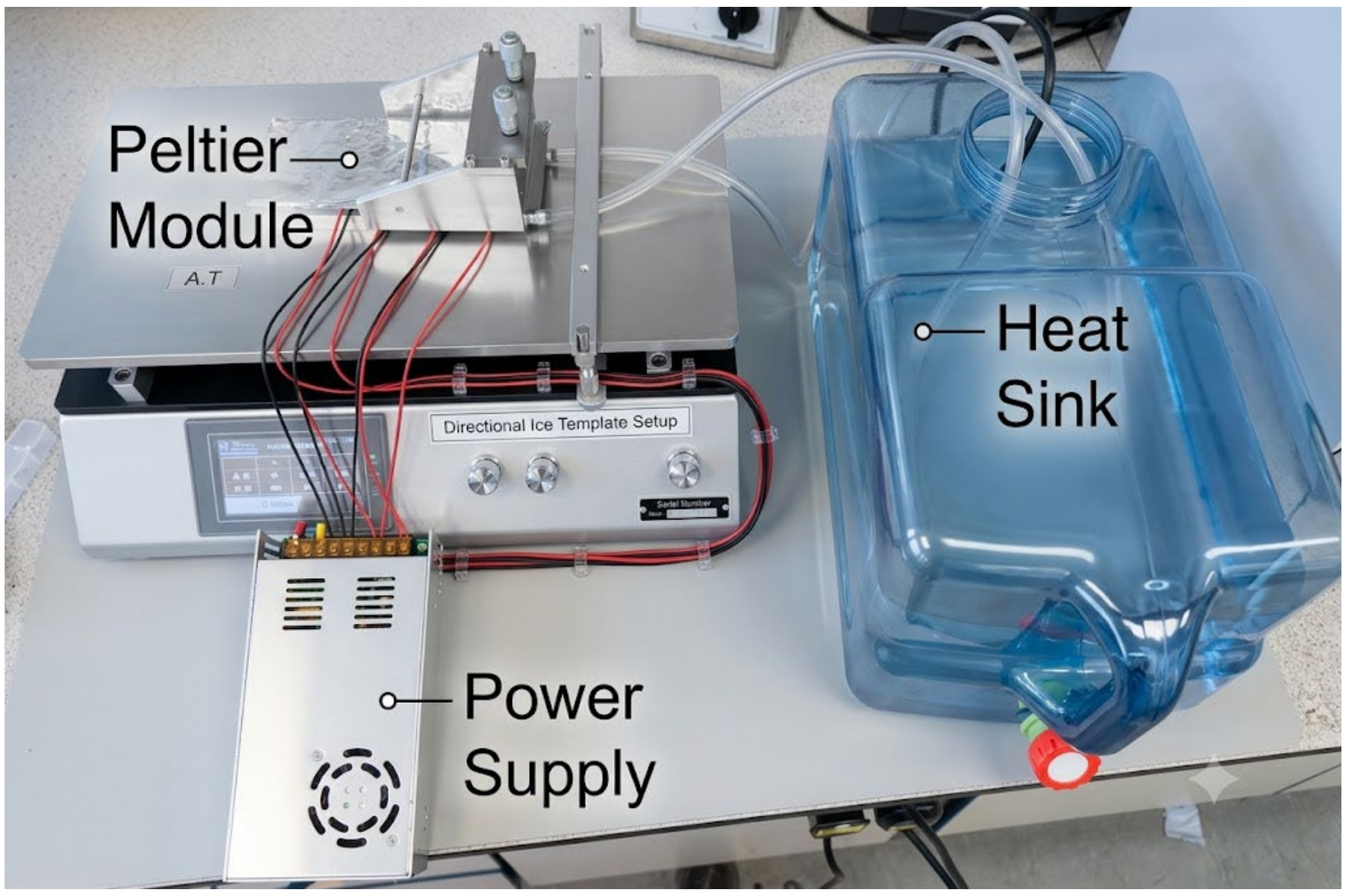

Figure S4. Photograph of the experimental directional ice-templating (DIT) setup

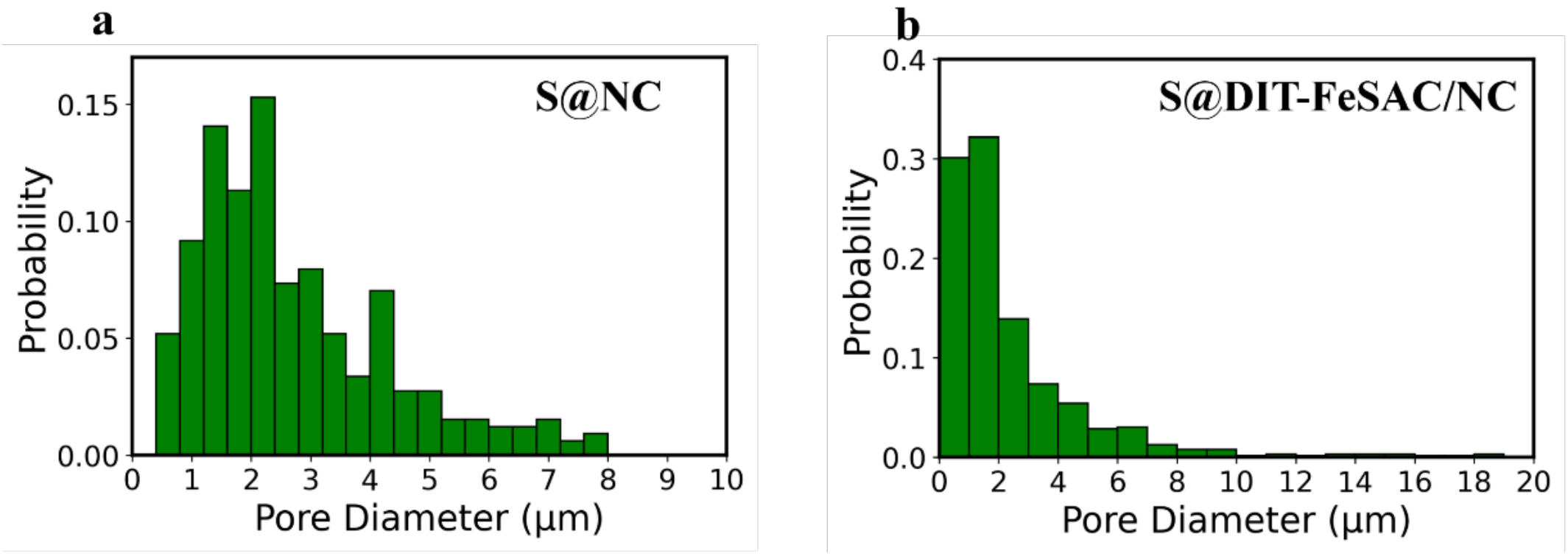

Figure S5. Pore size distribution of a) S@NC and b) S@DIT-FeSAC/NC

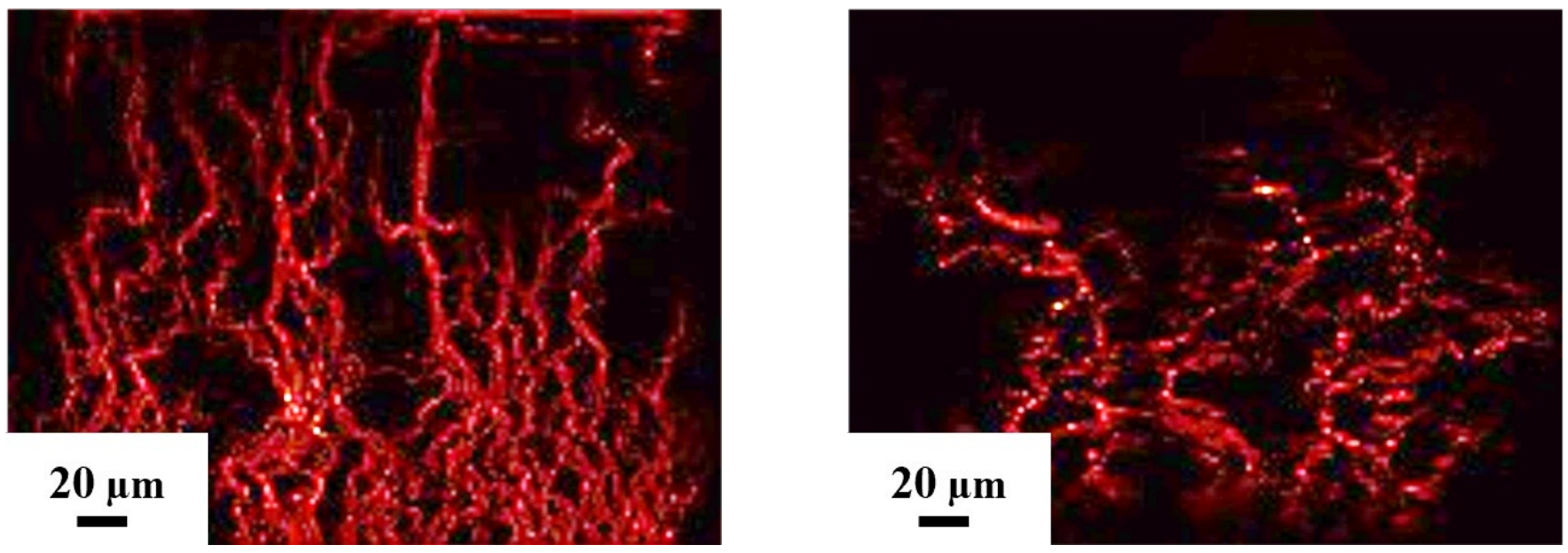

Figure S6. Simulated through-thickness flux-density maps derived from segmented cross-sectional SEM (a) S@DIT-FeSAC/NC cathode, (b) S@NC cathode.

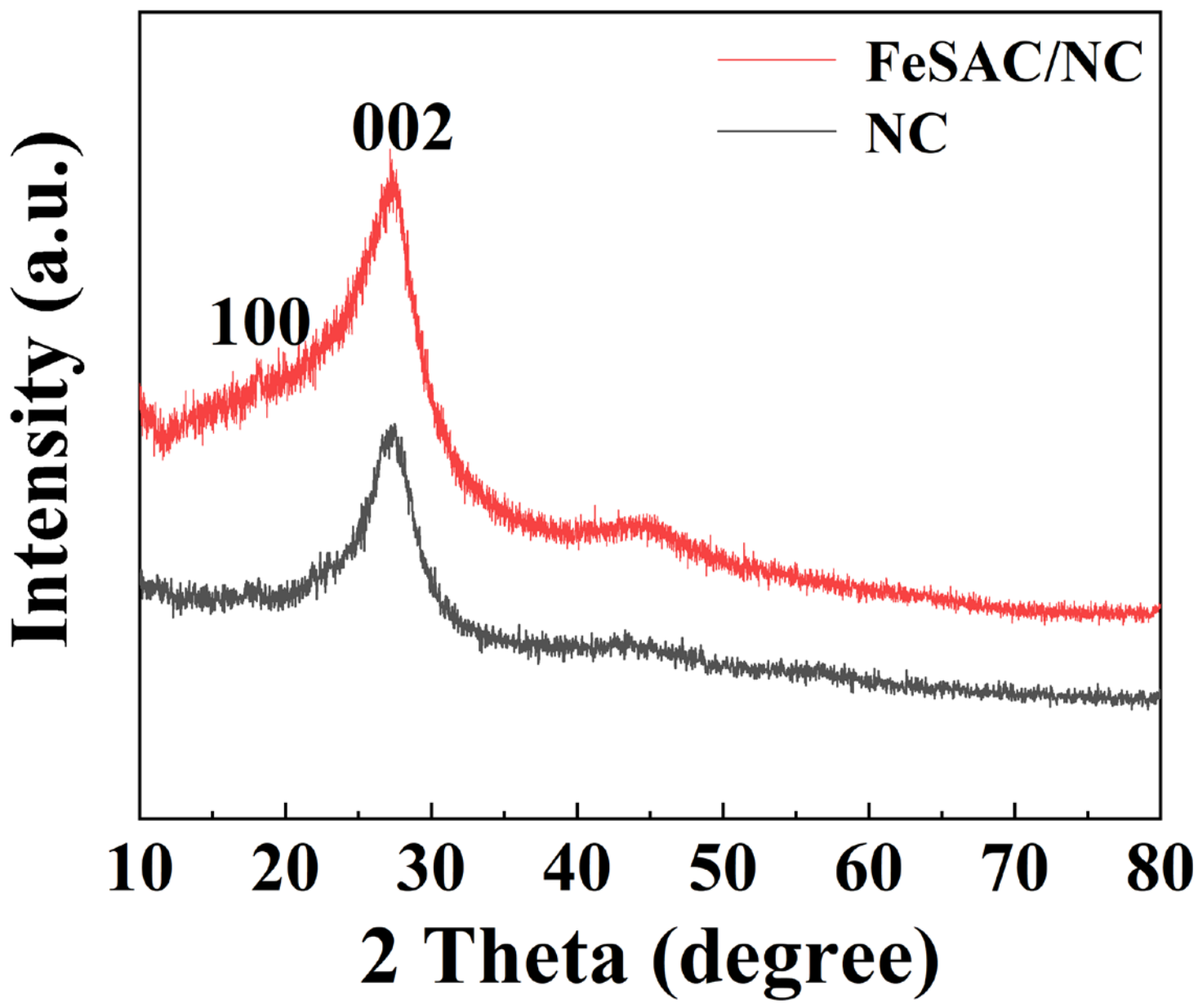

Figure S7. XRD patterns of FeSAC/NC and NC in powder form.

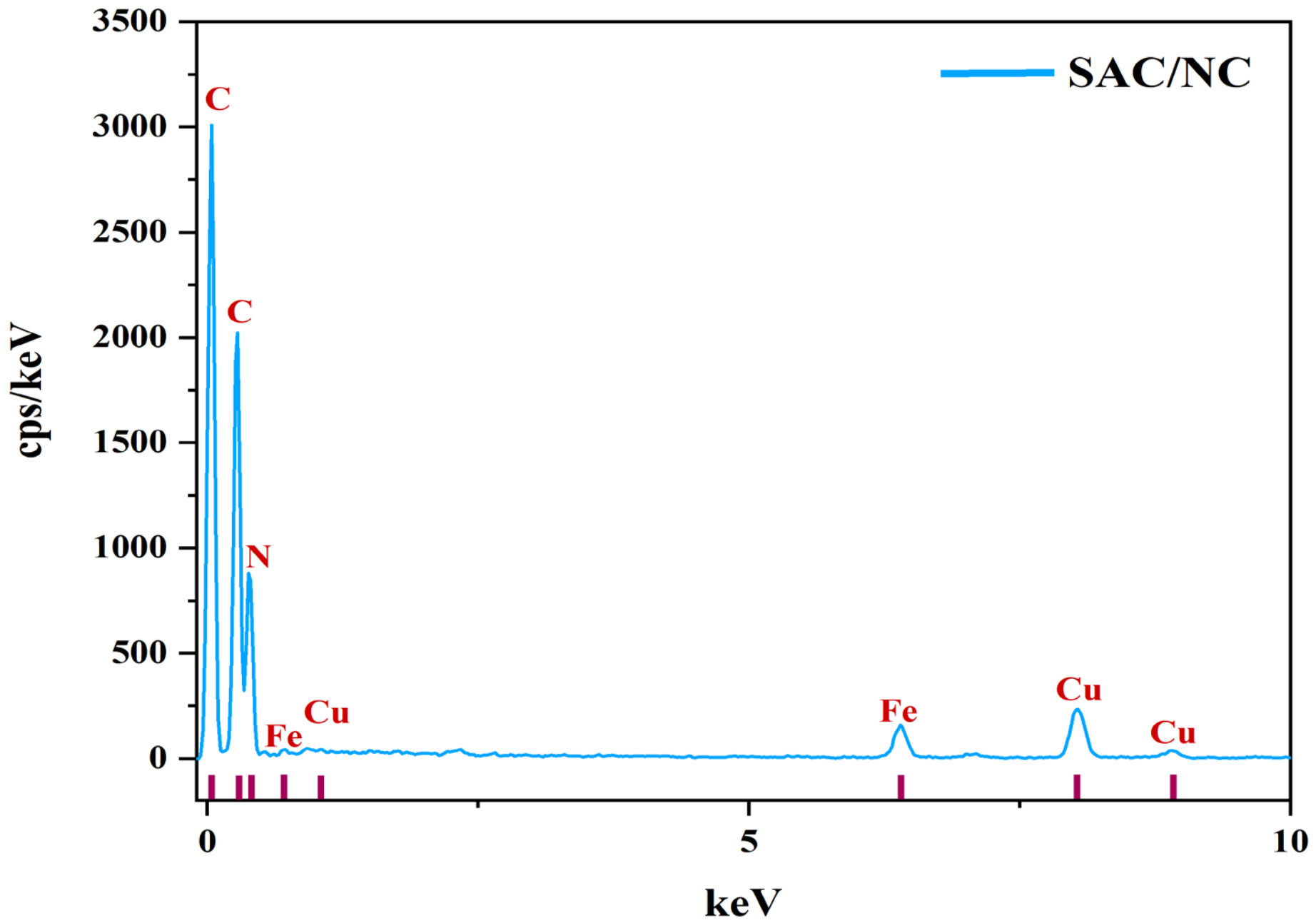

Figure S8. EDS of FeSAC/NC through TEM.

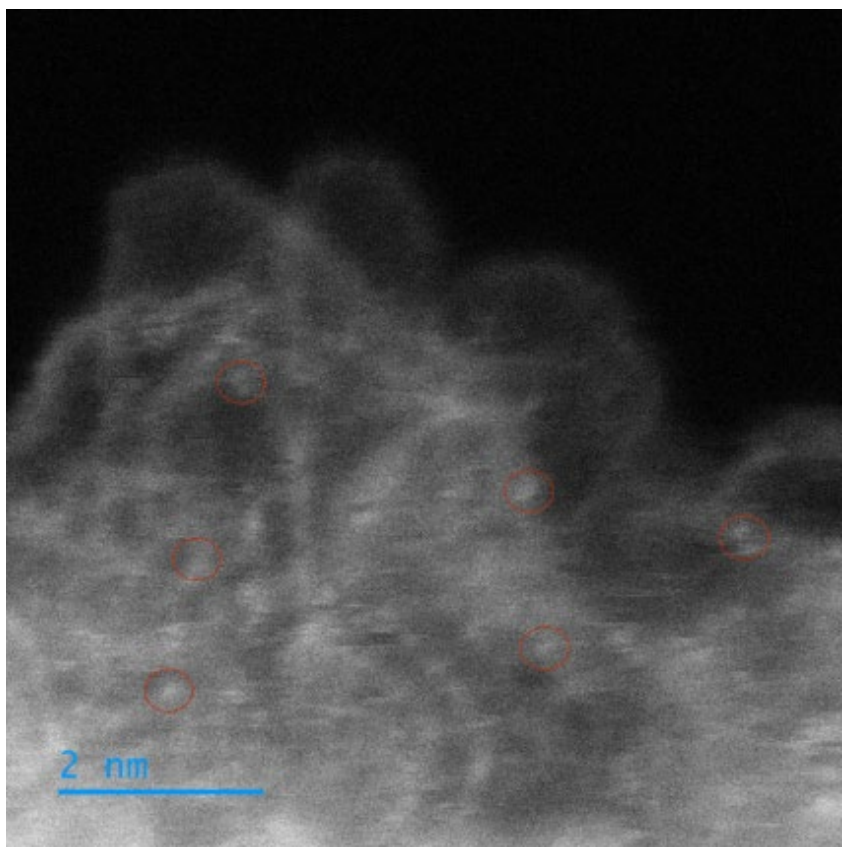

Figure S9. HAADF-STEM image of FeSAC/NC.

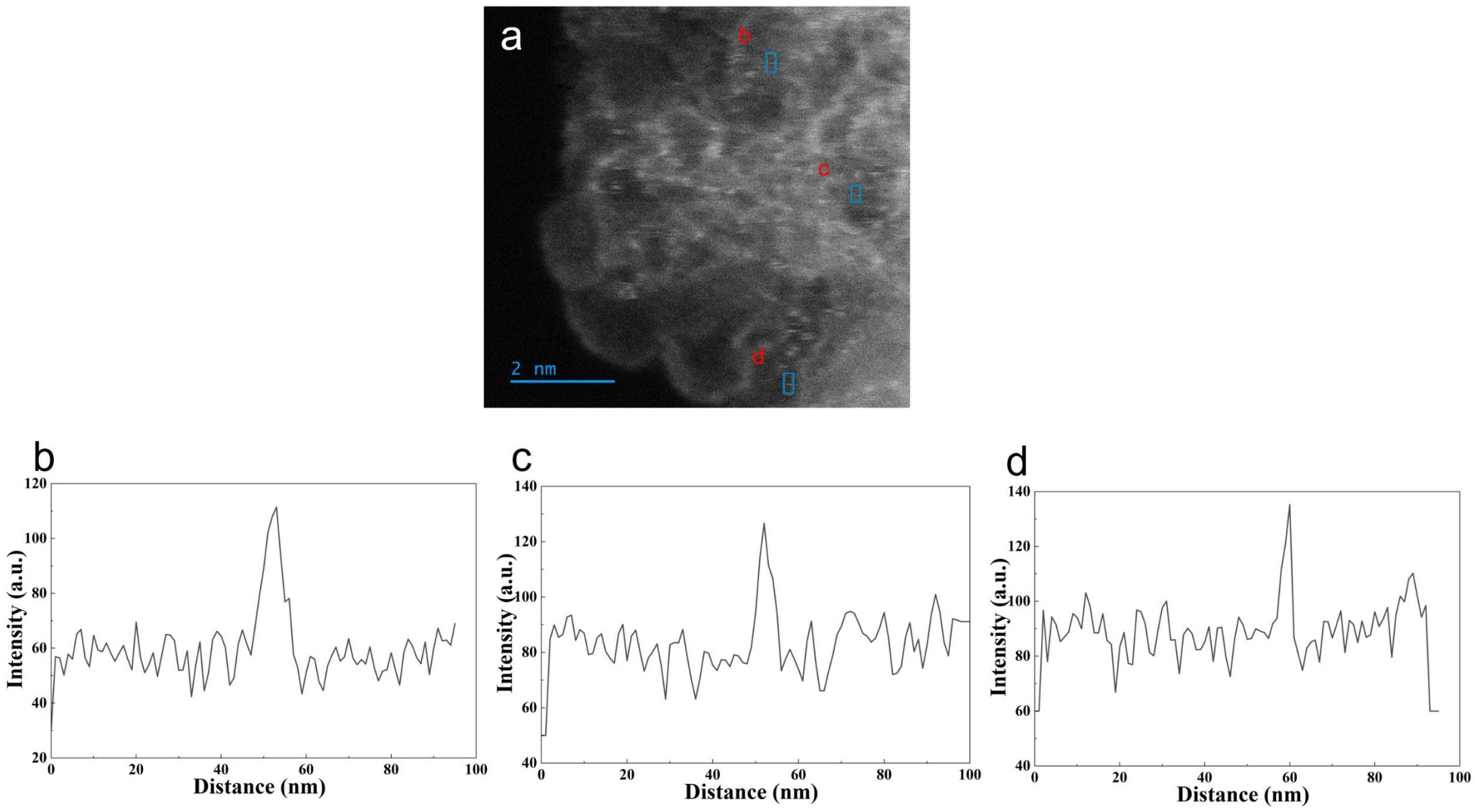

Figure S10. (a) HAADF-STEM image of FeSAC/NC; and (b-d) the relating intensity profiles extracted across three representative isolated bright spots.

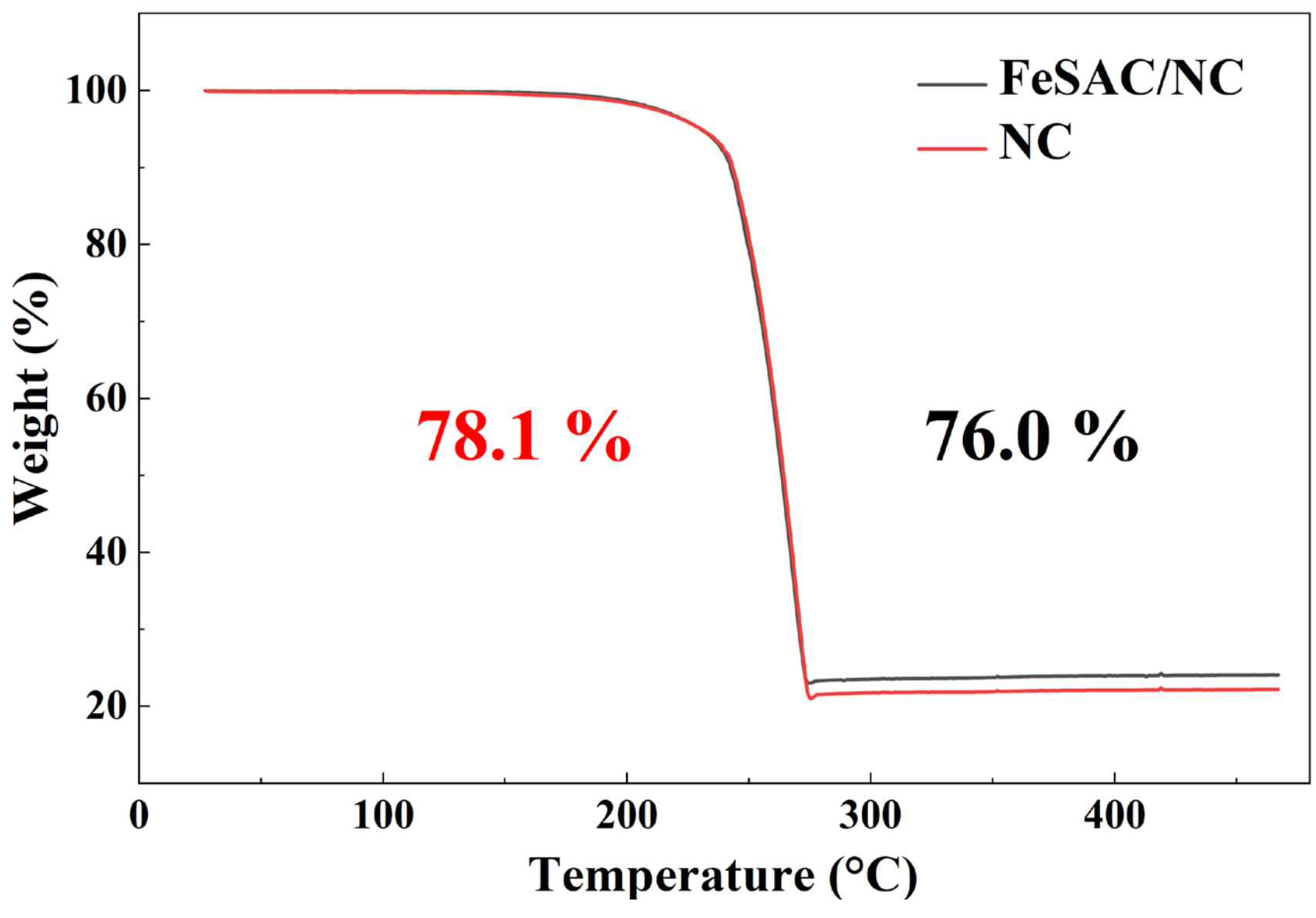

Figure S11. Thermogravimetric (TGA) analysis of S@FeSAC/NC and S@NC.

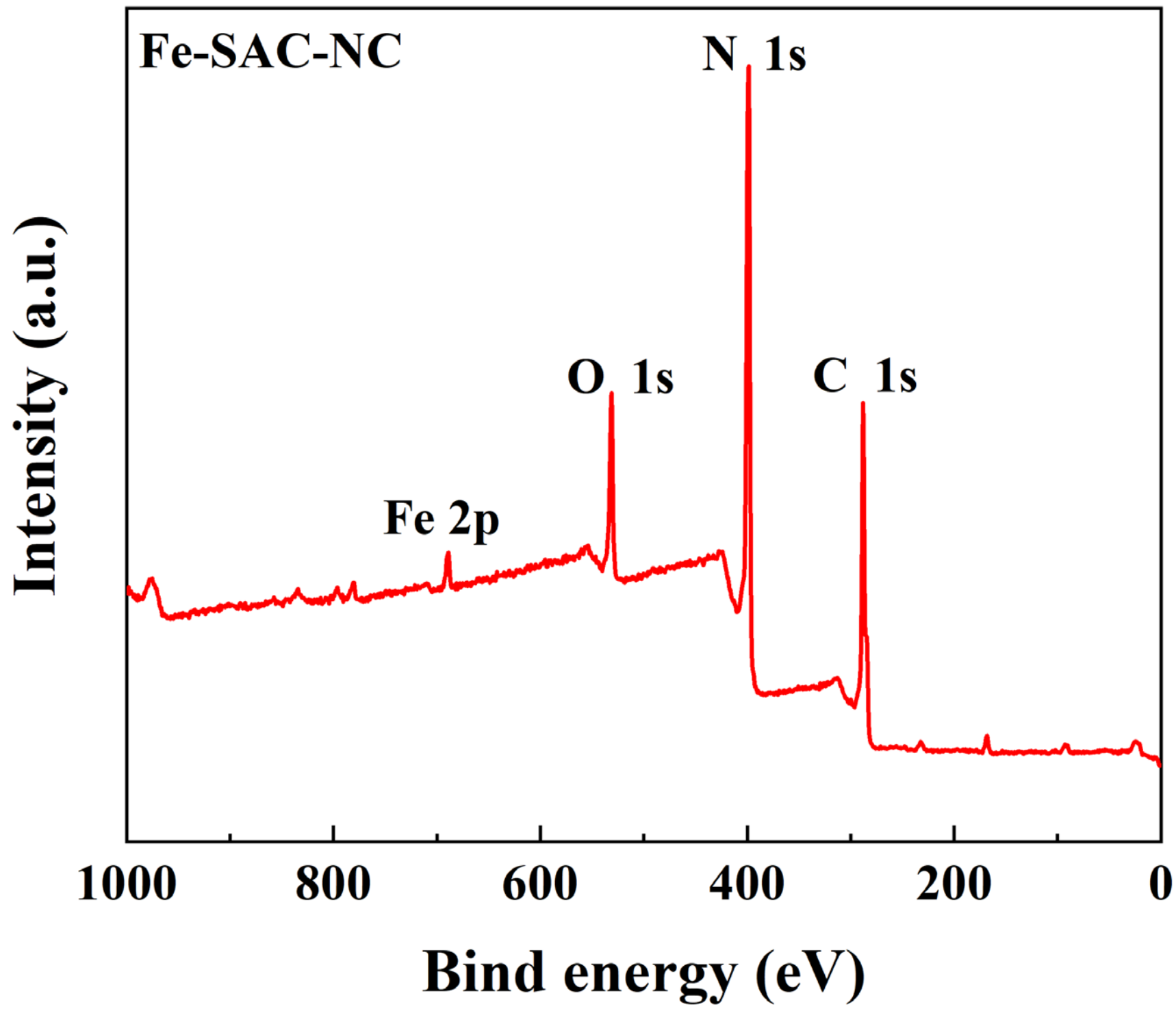

Figure S12. XPS full scan of FeSAC/NC.

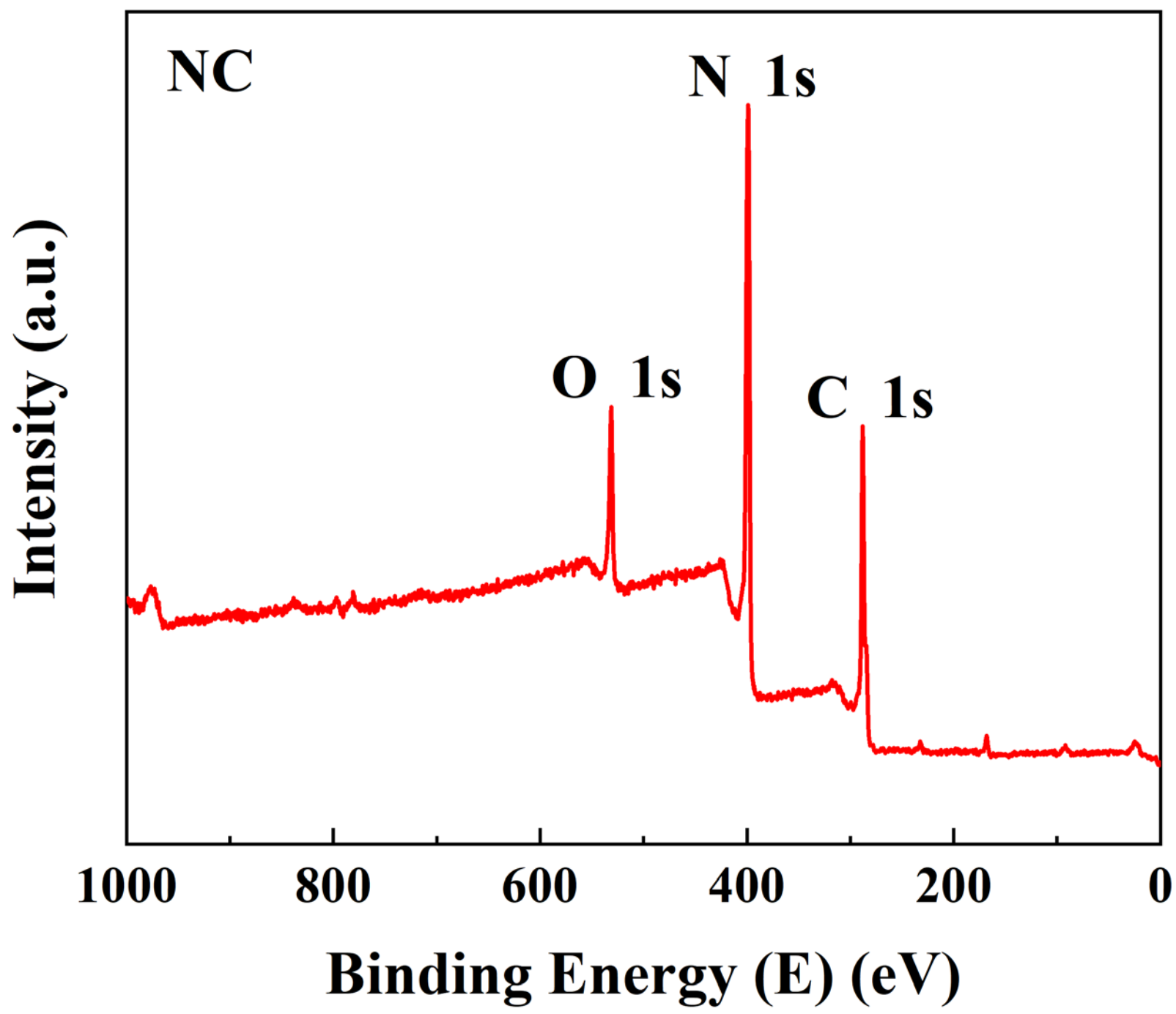

Figure S13. XPS full scan of NC.

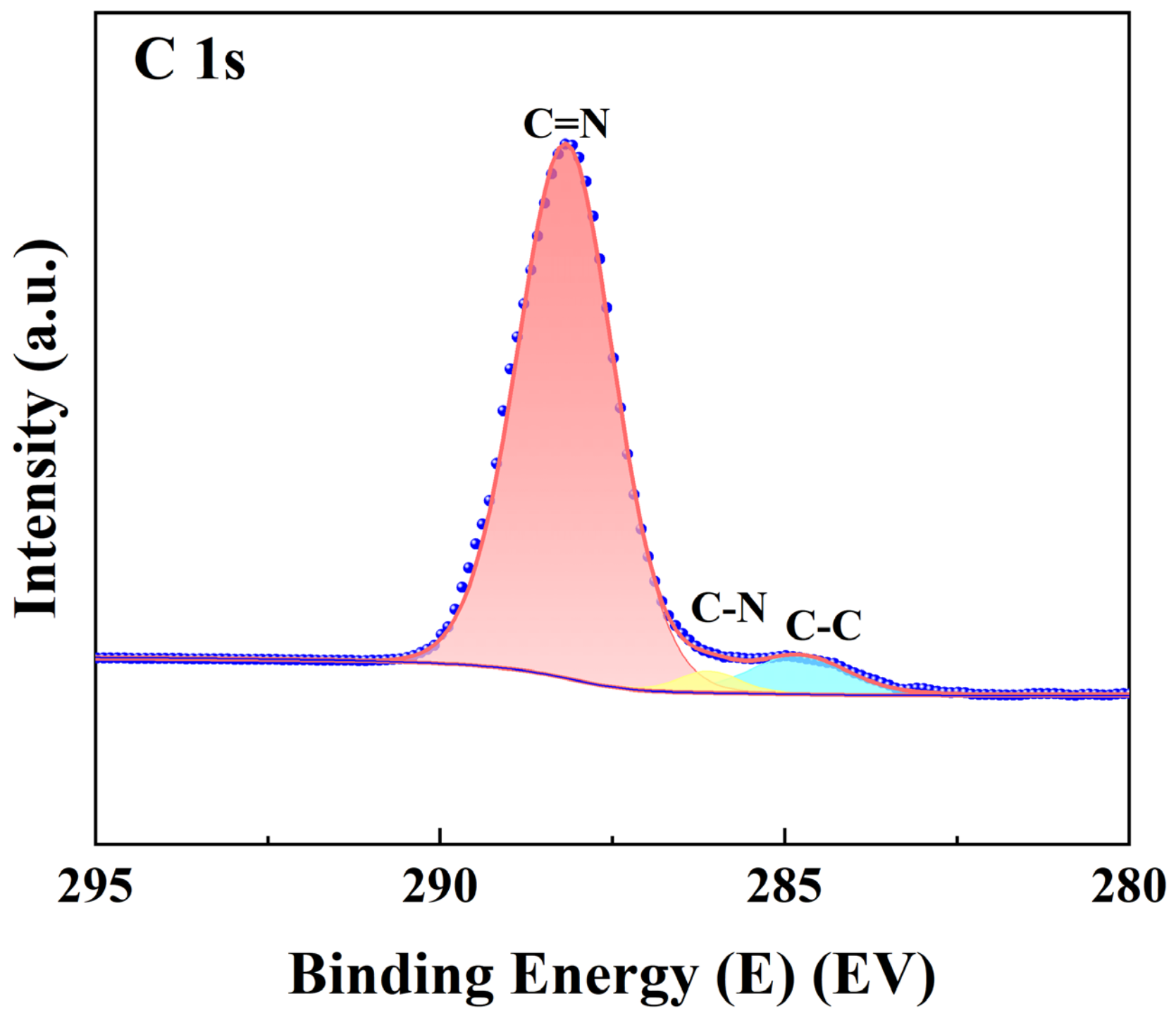

Figure S14. XPS of C 1s spectrum for NC.

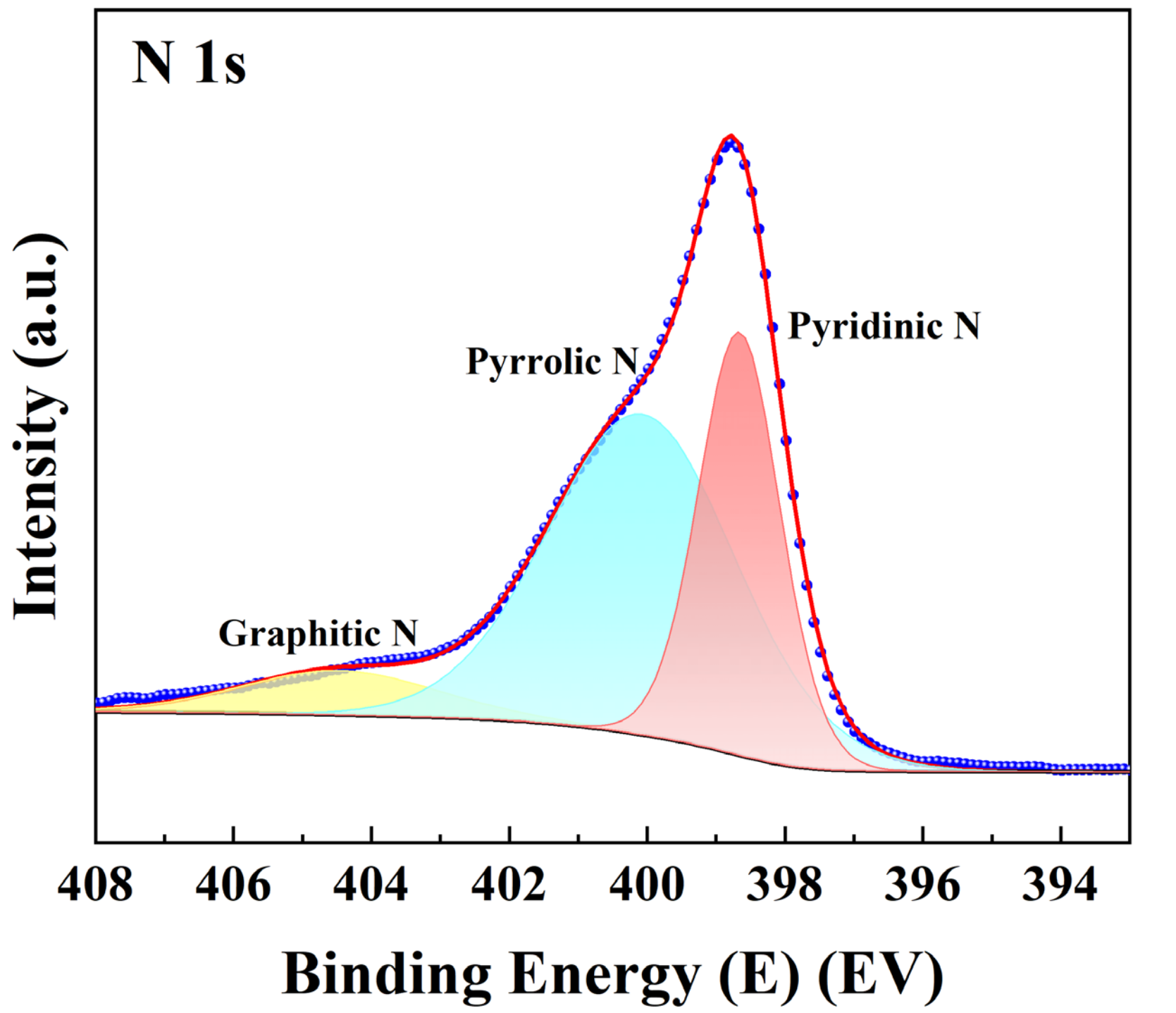

Figure S15. XPS of N 1s spectrum for NC.

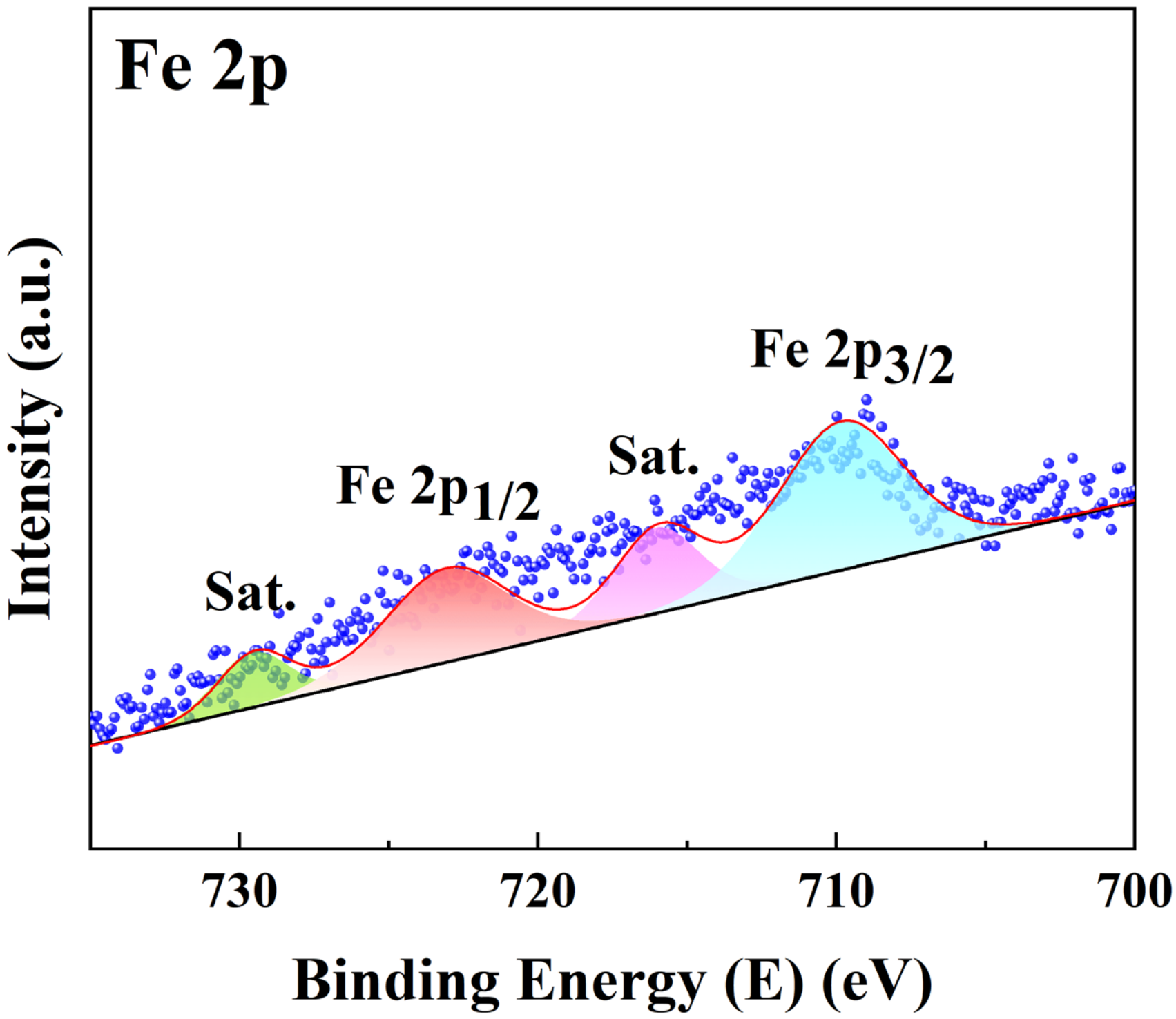

Figure S16. XPS of Fe 2p spectrum for FeSAC/NC.

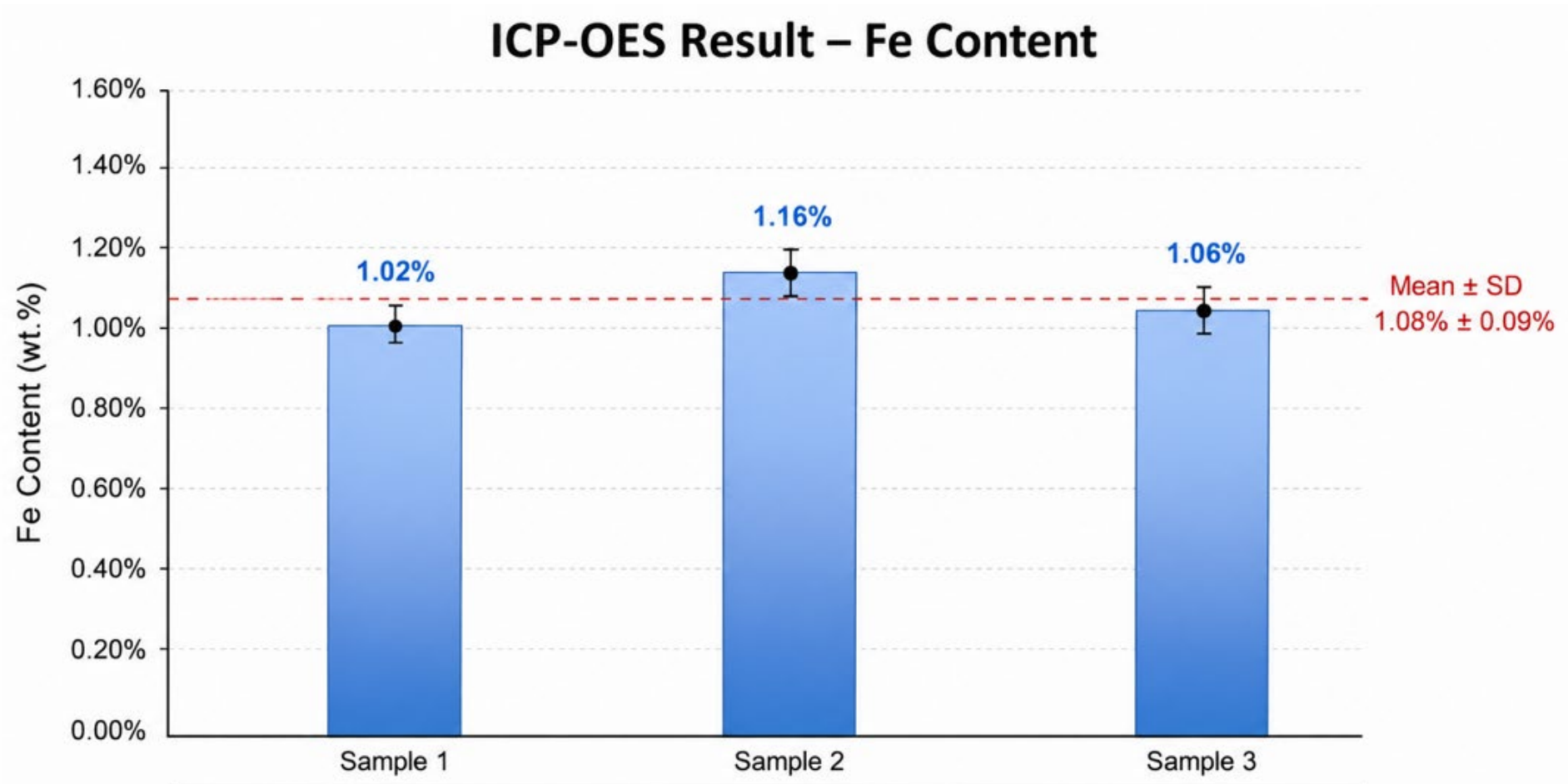

Sample	Fe Content (wt.%)
Sample 1	1.02
Sample 2	1.16
Sample 3	1.06
**Mean ± SD**	**1.08% ± 0.09%**

Method: ICP_OES
Unit: wt.% (w/w)

Figure S17. ICP-OES results of FeSAC/NC.

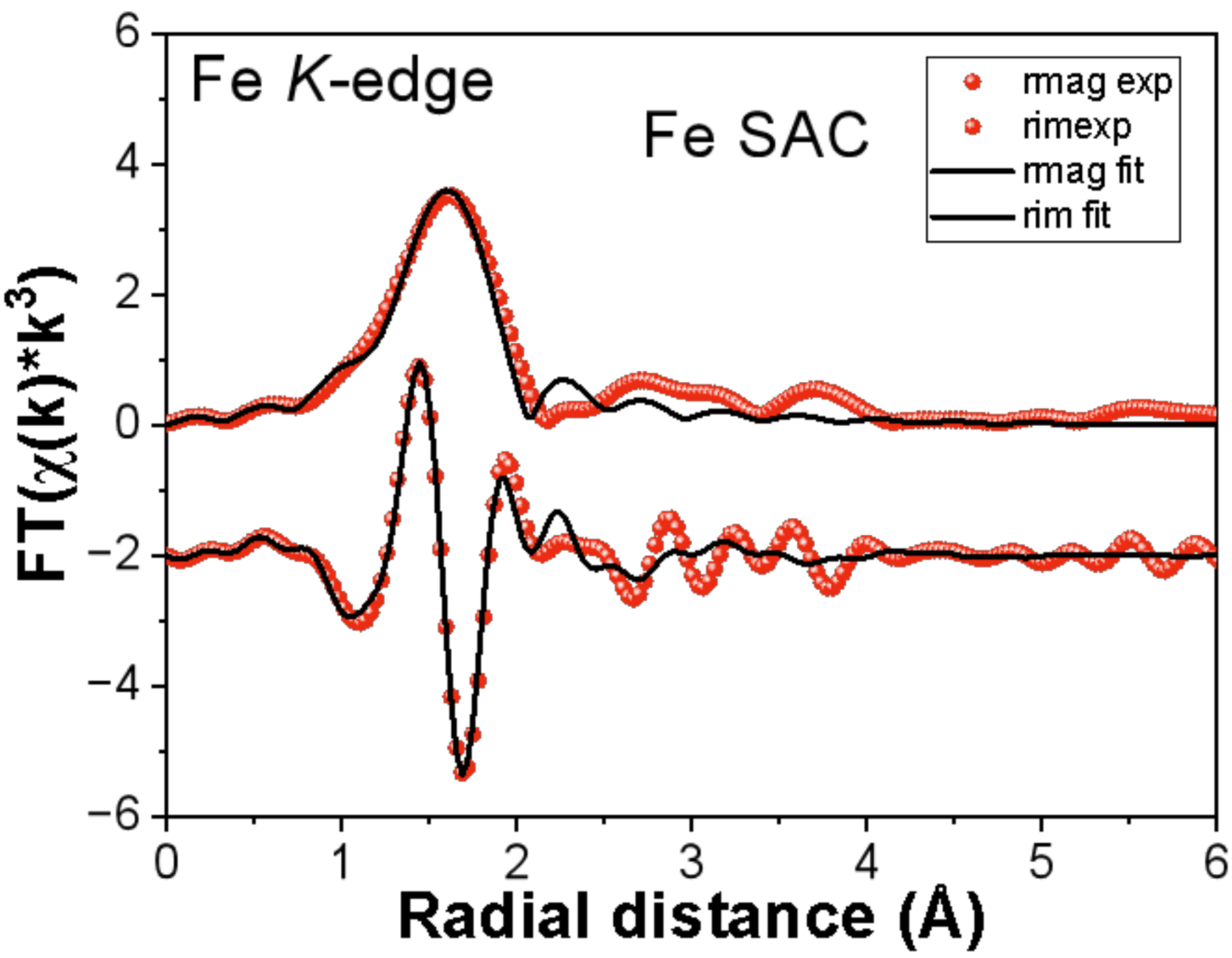

Sample	Shell	CN	R(Å)	σ²(Å²)	ΔE₀(eV)	R factor
Fe SAC	Fe-N	4.31±0.71	2.09±0.01	0.0078±0.0028	2.0830±0.0057	0.0160

Figure S18. EXAFS fitting parameters at the Fe K-edge for Fe SAC samples.

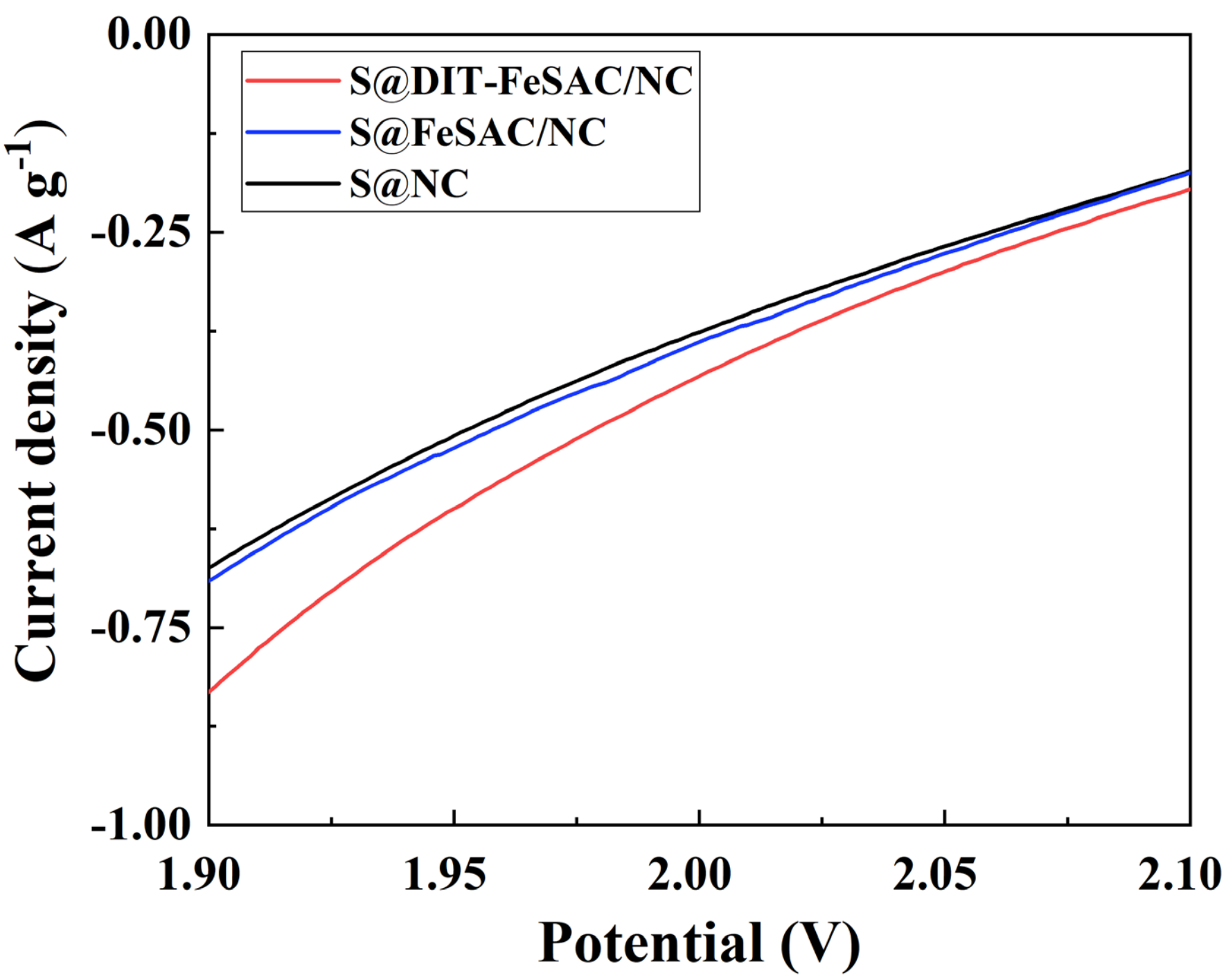

Figure S19. Linear scanning voltammetry (LSV) of reduction process.

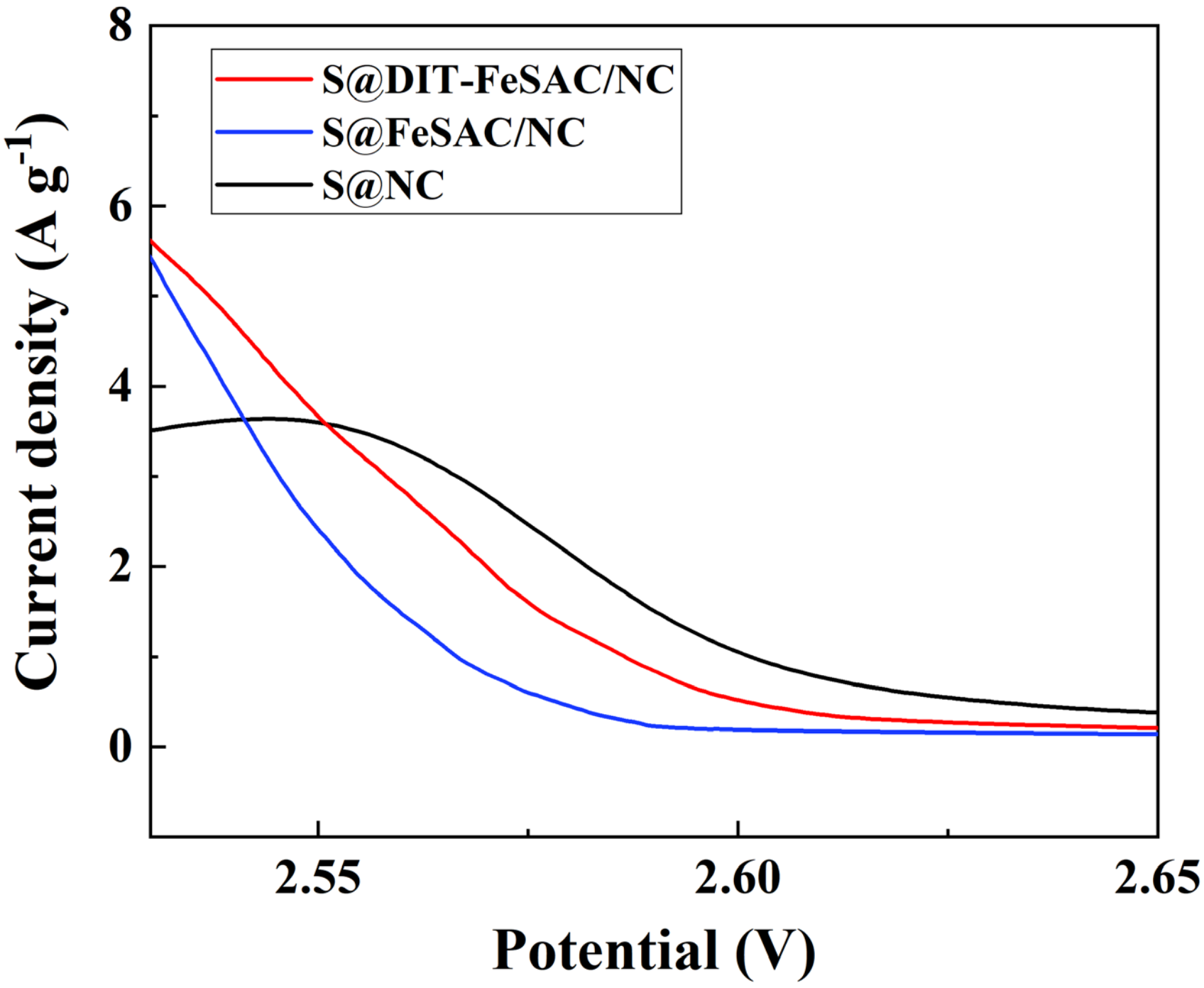

Figure S20. Linear scanning voltammetry (LSV) of oxidation process.

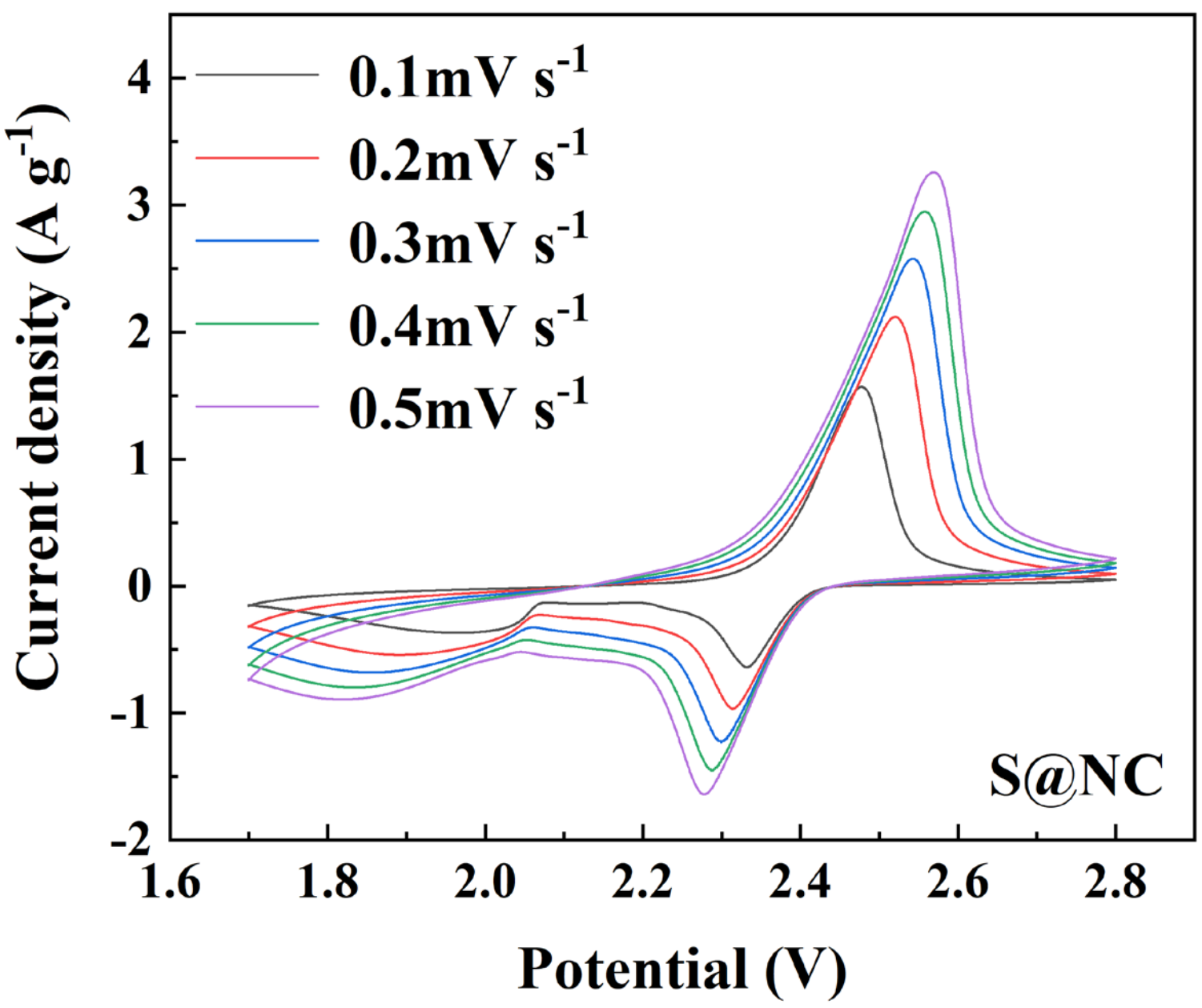

Figure S21. CV curves of the S@NC cathodes vs Li metal anode with different scan rates from 0.1 to 0.5 mV s^{-1}.

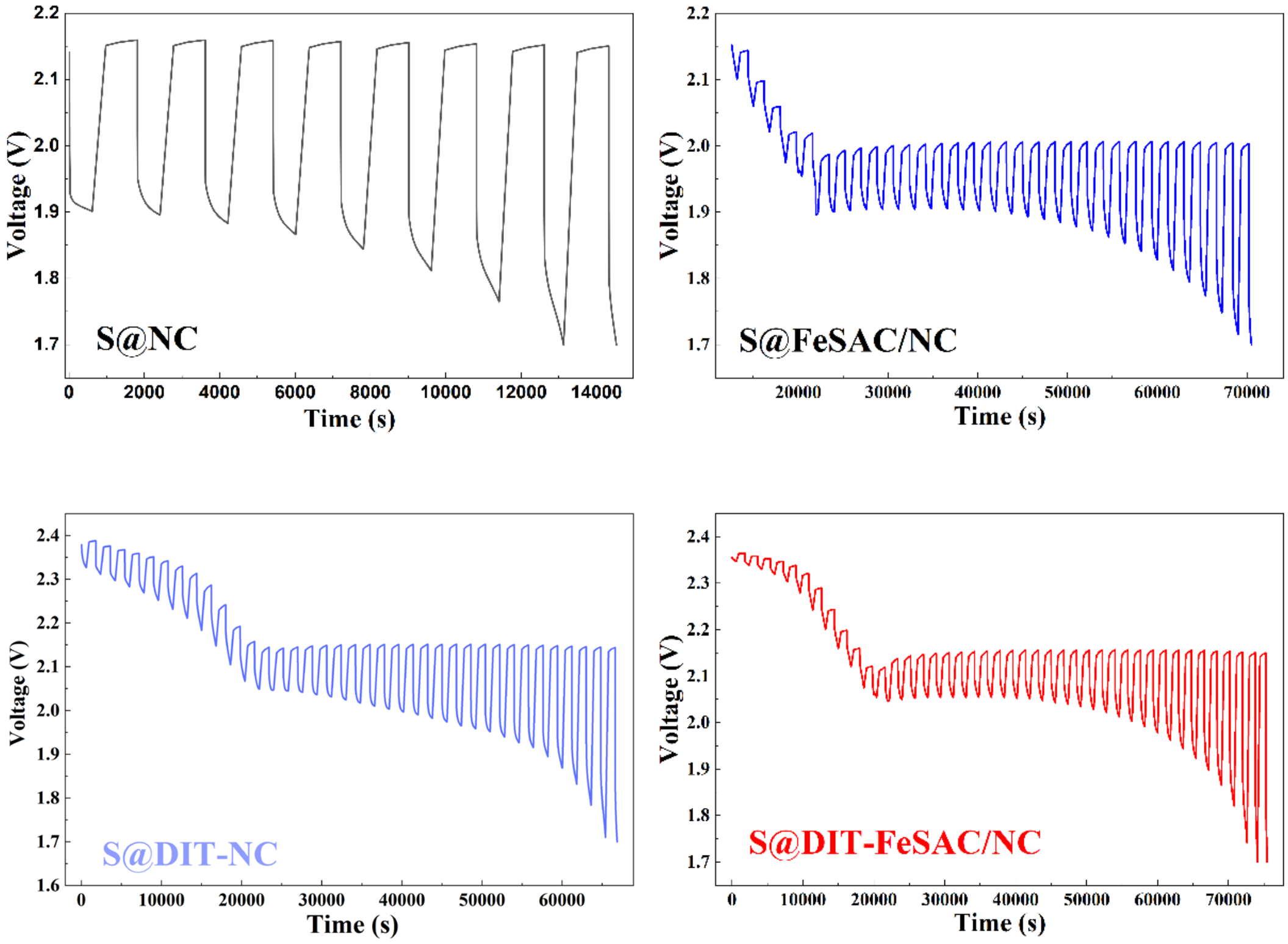

Figure S22. Galvanostatic intermittent titration technique (GITT) of S@NC, S@FeSAC/NC, S@DIT-NC and S@DIT-FeSAC/NC

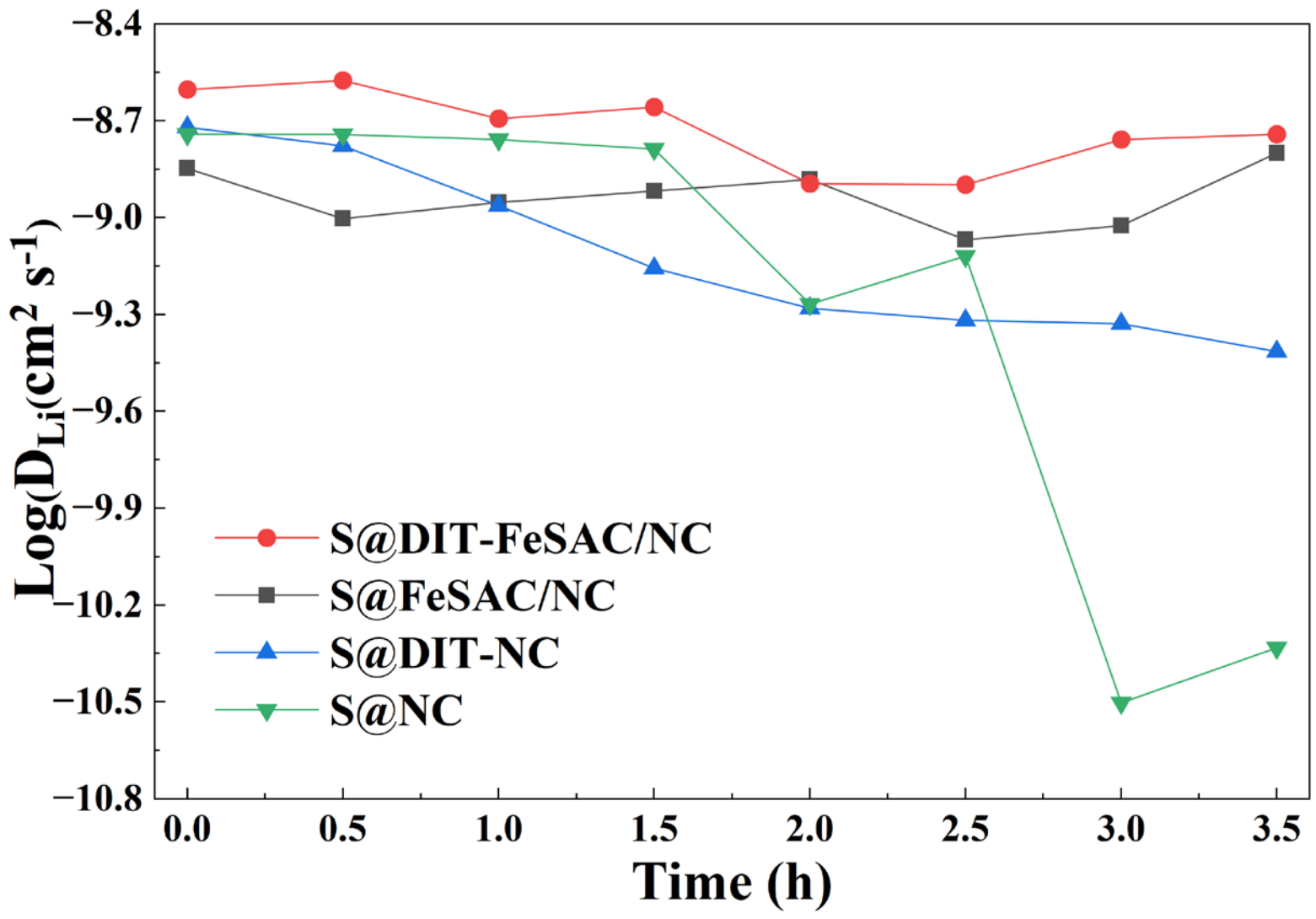

Figure S23. Comparison of the time-dependent Li-ion diffusion coefficients.

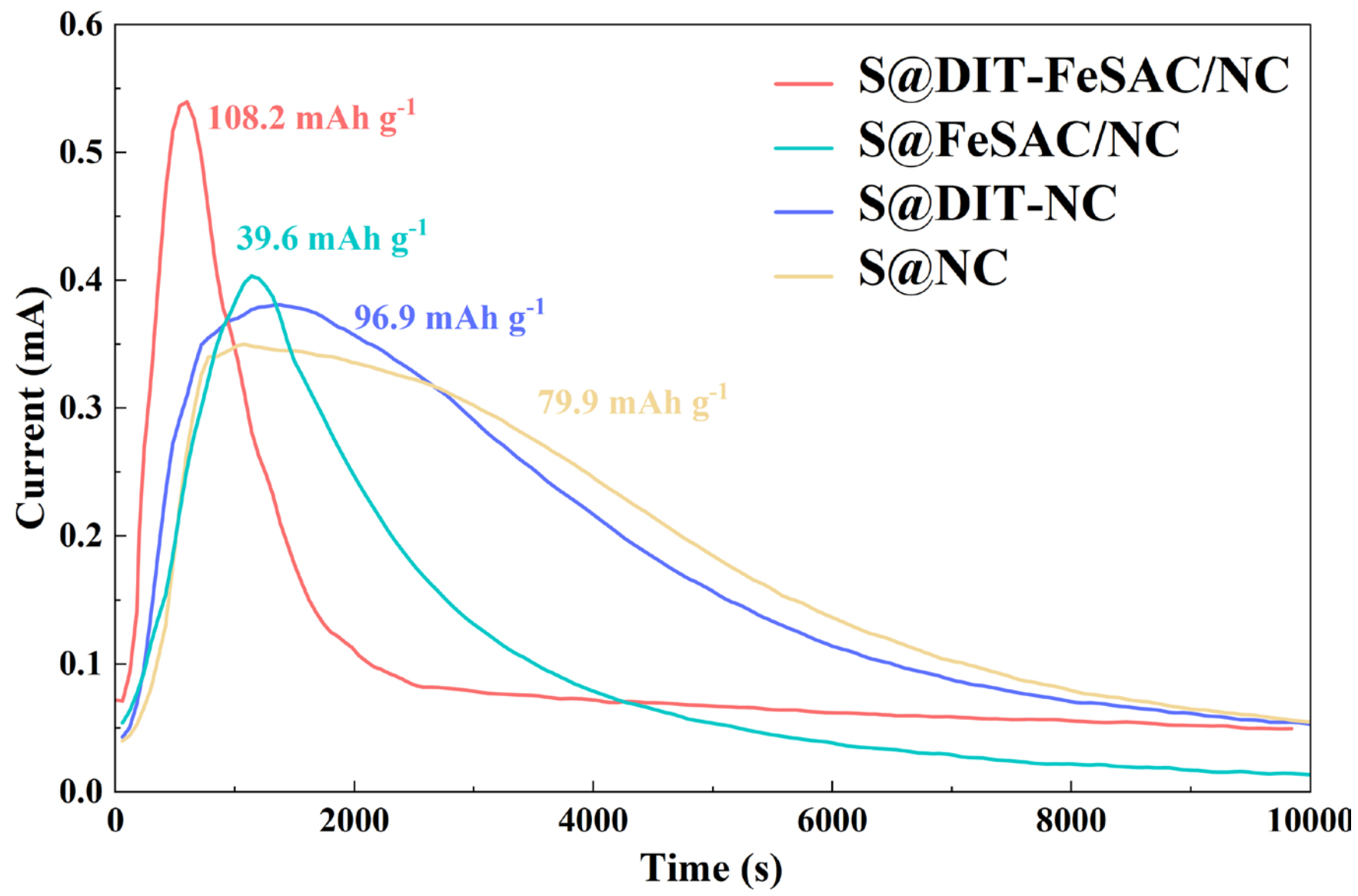

Figure S24. Li_2S nucleation behavior of S@NC, S@FeSAC/NC, S@DIT-NC and S@DIT-FeSAC/NC electrodes evaluated by chronoamperometry at a constant potential of 2.05 V.

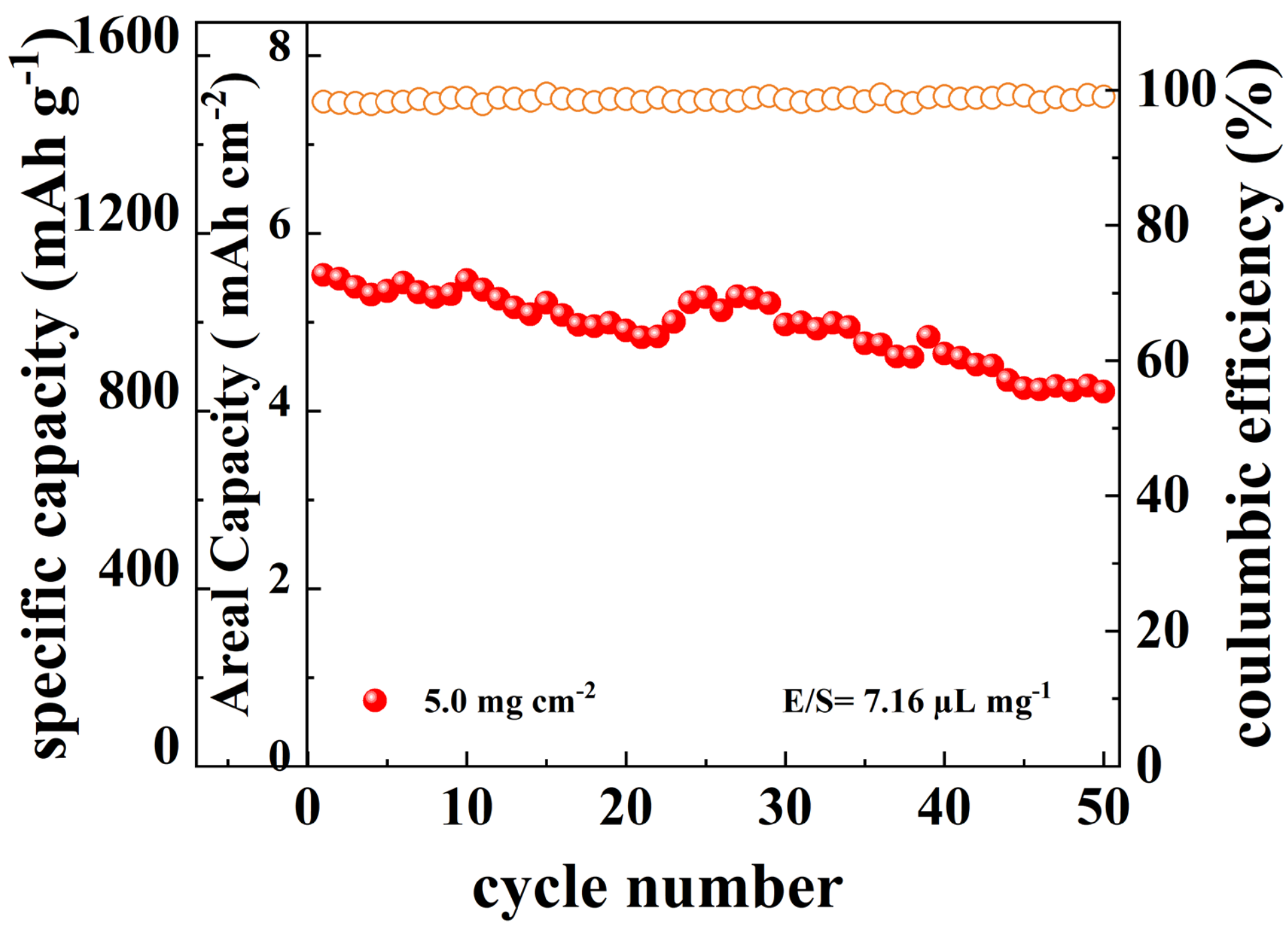

Figure S25. Cycling performances of the S@DIT-FeSAC/NC electrode with a sulfur loading of 5.0 mg cm^{-2}.

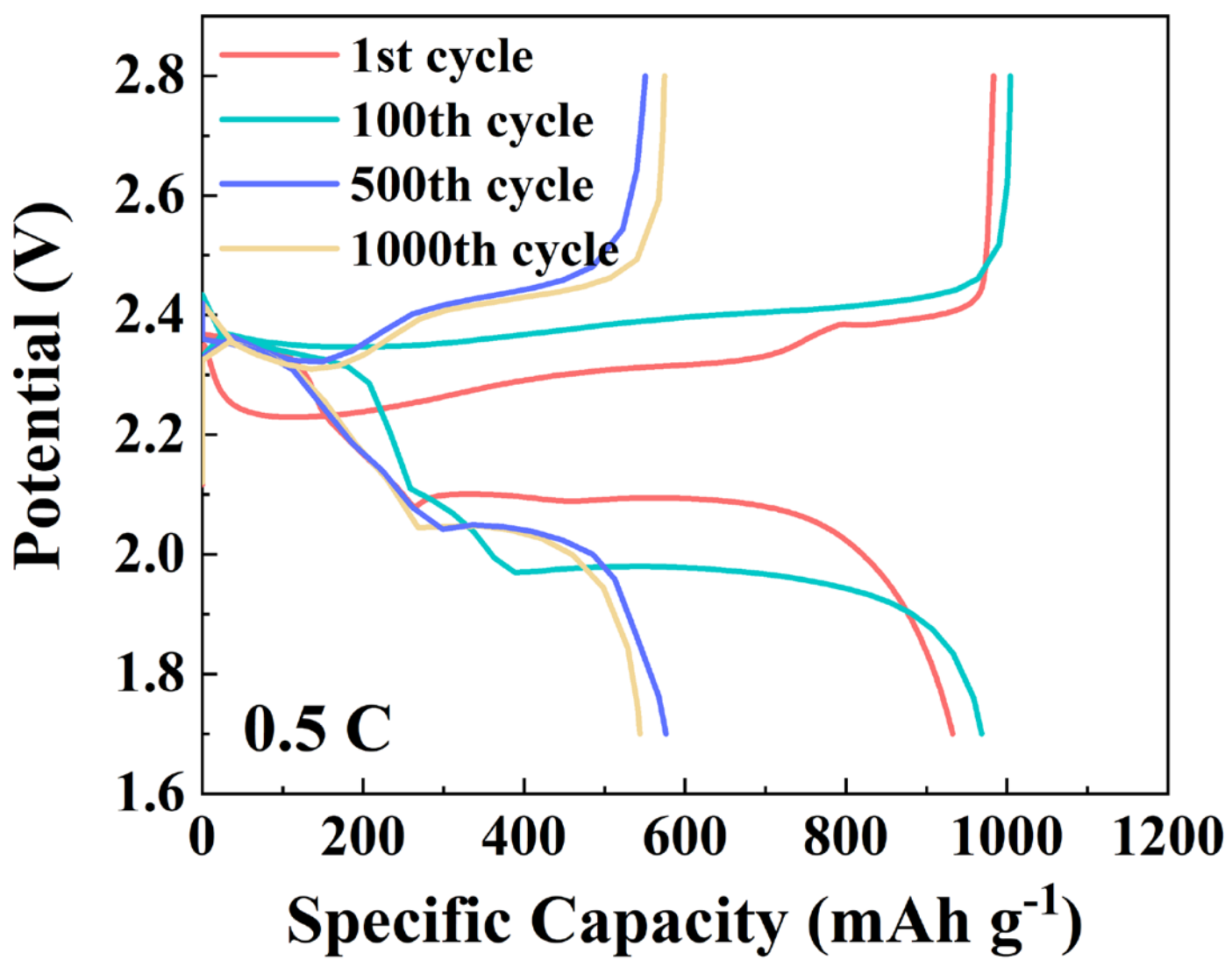

Figure S26. Representative galvanostatic charge–discharge voltage profiles of the S@DIT-FeSAC/NC cathode at the 1st, 100th, 500th, and 1000th cycles measured at 0.5 C.

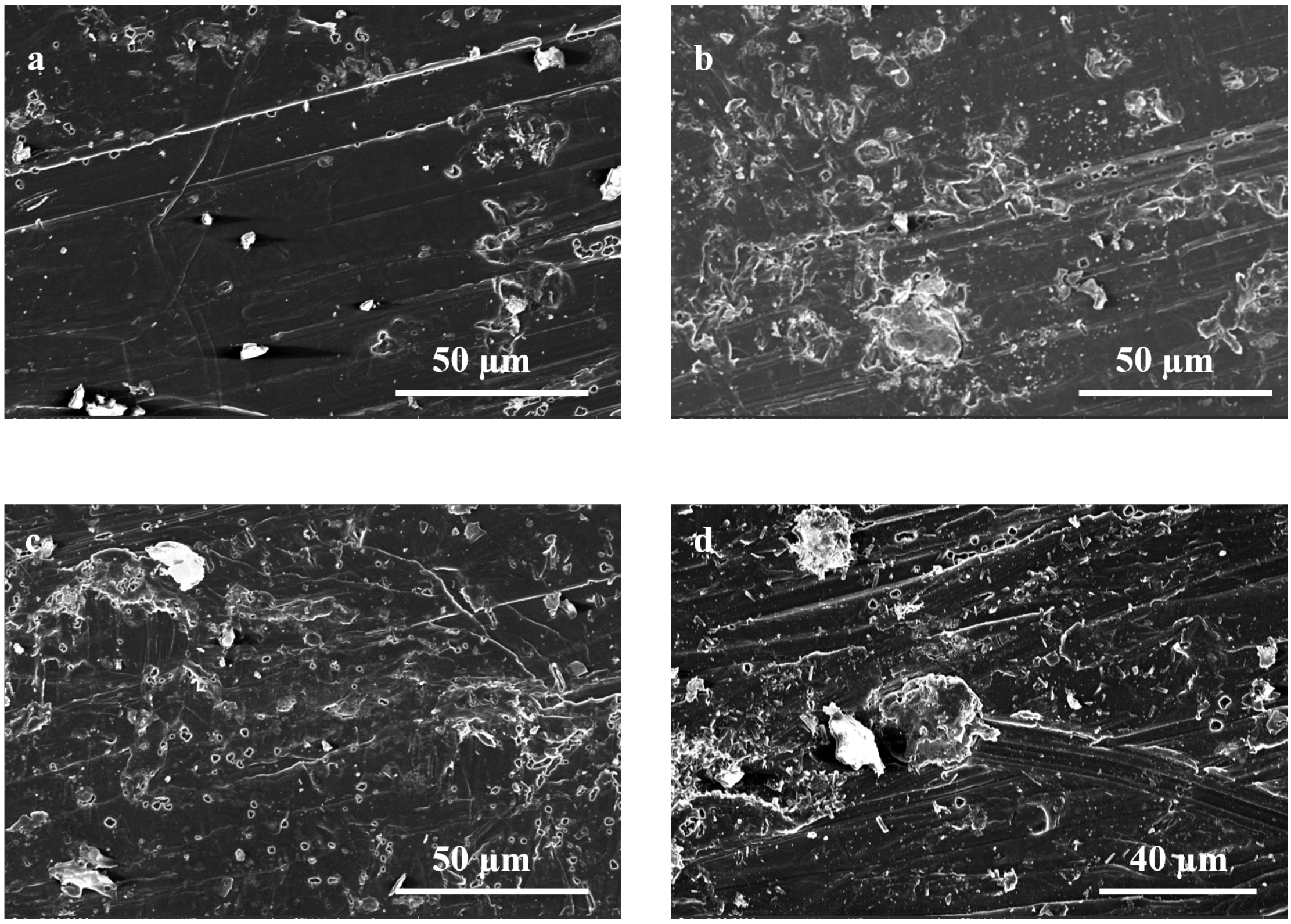

Figure S27. SEM images of the Li metal anodes from cells after 100 cycles. The cathodes are (a) S@DIT-FeSAC/NC, (b) S@DIT-NC, (c) S@FeSAC/NC, and (d) S@NC.

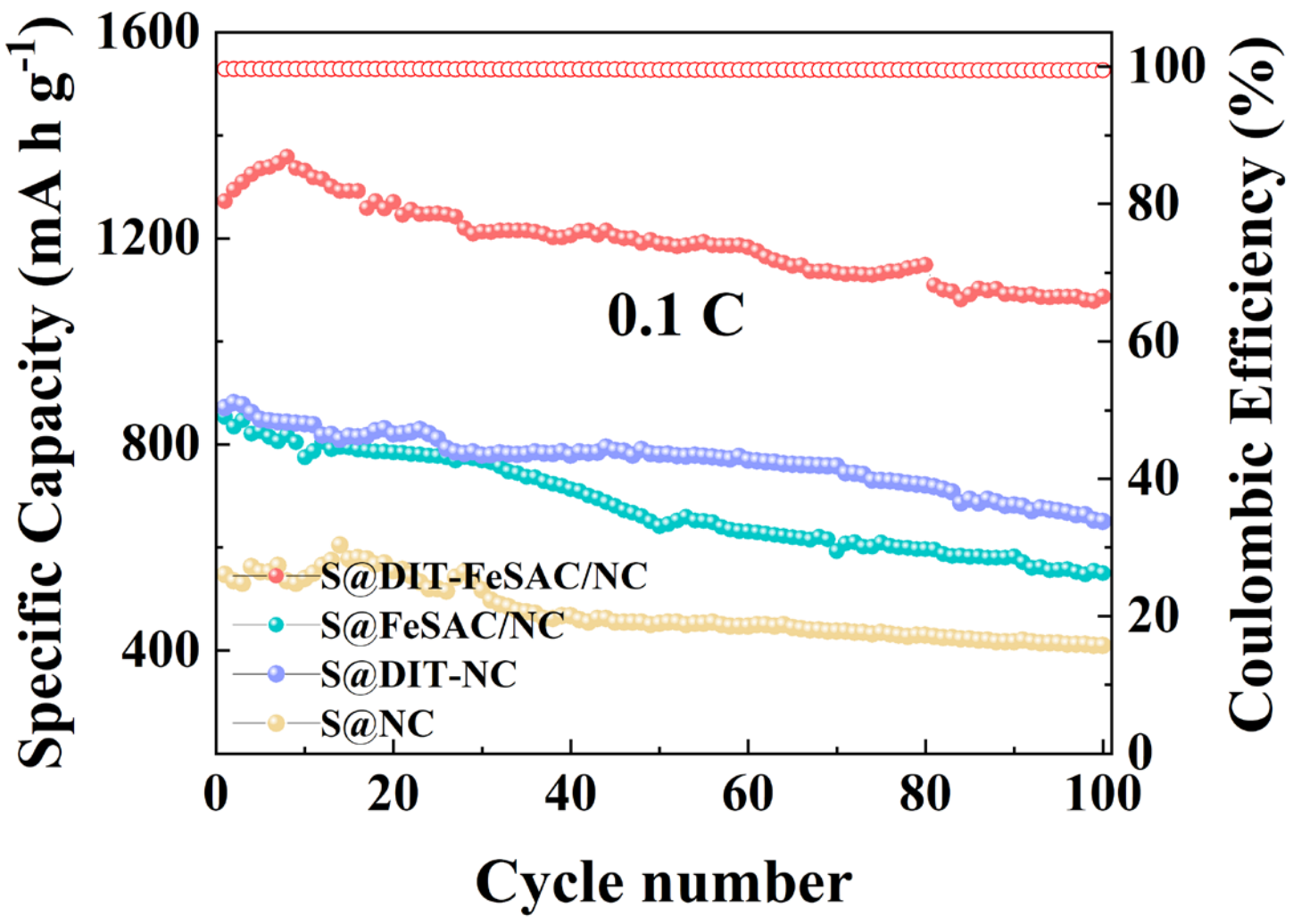

Figure S28. Representative repeat measurements of the 0.1 C cycling performance.

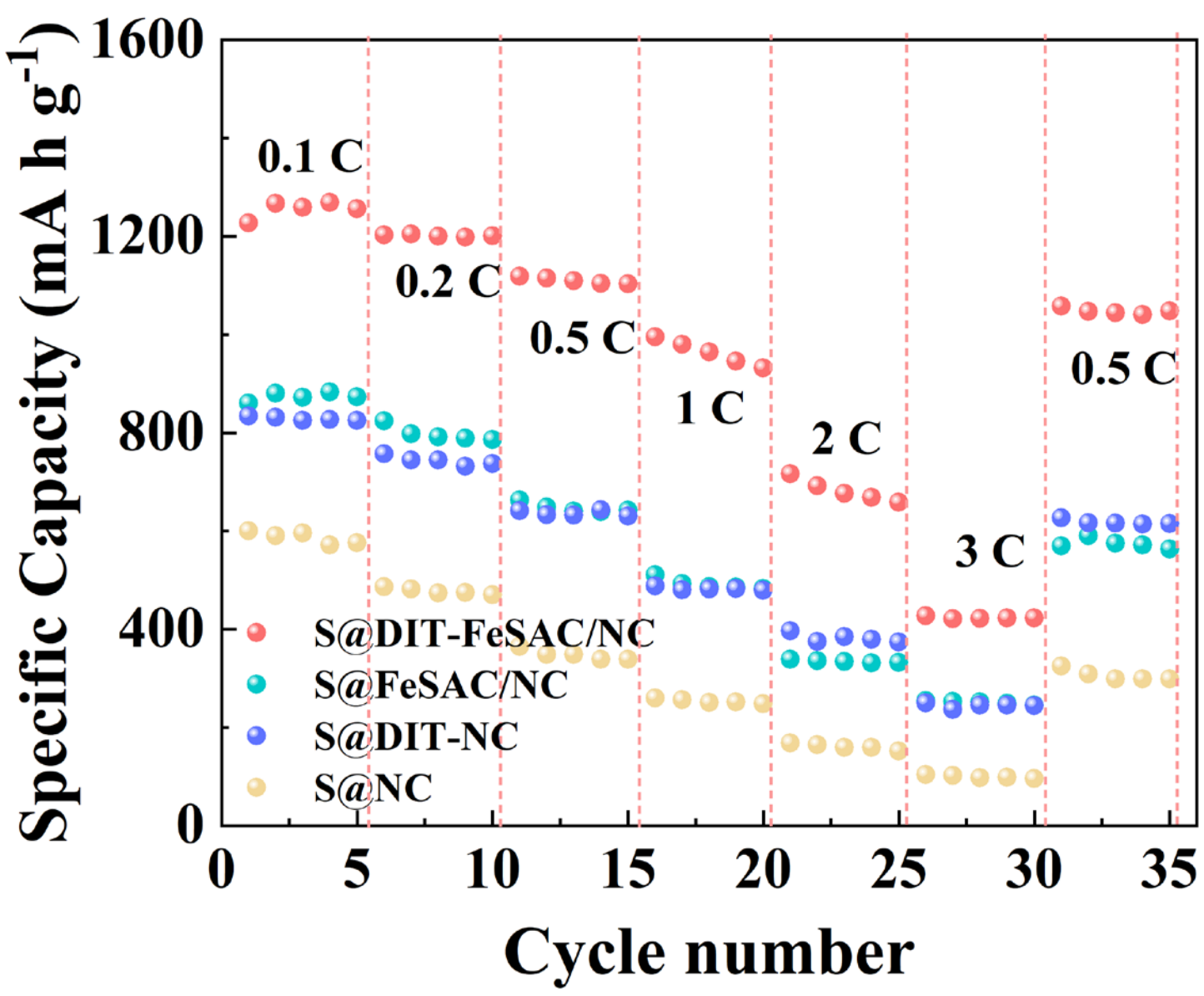

Figure S29. Representative repeat measurements of the rate capability.

Table S1. Comparison of the electrochemical performances of representative state-of-the-art single-atom catalysts (SACs) together with representative non-single-atom catalysts reported for Li–S batteries.

Single atoms	Capacity	Sulfur loading (mg cm^{-2})	E/S	Long cycle	Decay rate	Reference
DIT-FeSAC/NC	1299.2mAh g^{-1} at 0.1C	2.0	7.16	505.9 mAh g^{-1} after 1000 cycles at 0.5C	0.042%	This work
SA-Fe/Fe2N@NG	1457.3 mAh g^{-1} at 0.1 C	1.5	20	801.3 mAh g^{-1} after 500 cycles at 1C	0.032%	[7]
Fe-N5-C	1224 mAh g^{-1} at 0.2 C	1.0	19.1	662 mAh g^{-1} after 500 cycles at 1C	0.054%	[8]
Ni−N5/HNPC	1188 mAh g^{-1} at 0.2C	1.6	14	798 mAh g^{-1} after 500 cycles at 0.5C	0.053%	[10]
Co-NG(800)	1346 mAh g^{-1} at 0.1C	1.0	30	505 mAh g^{-1} after 600 cycles at 0.5C	0.08%	[11]
NC@SA-Co	1160 mAh g^{-1} at 0.1C	1.0	40	467 mAh g^{-1} after 700 cycles at 2 C	0.058%	[12]
Co-N-C	1247mAh g^{-1} at 0.2C	1.0	18	850 mAh g^{-1} after 300 cycles at 0.5 C	0.1%	[13]
FeSA–CN	1123 mA h g^{-1} at 0.2C	2.4	20	605 mAh g^{-1} after 500 cycles at 4C	0.06%	[14]
$MoNi_x/Mo_2C$-NCNFs	1343.9mAh g^{-1} at 0.2 C	1.0	15	718.5 mAh g^{-1}after 1000 cycles at 1	0.024%	[15]

				C		
CoS_2–MgS	1156.1 mAh g^{-1} at 0.2 C	1.5	32	296.3 mAh g^{-1} after 600 cycles at 5 C	0.08%	[16]
Mo_2C–MoS_2	1208 mAh g^{-1} @ 0.2 C	1.2	18	444mAh g^{-1} after 1000 cycles @ 5 C	0.03%	[17]

References

1. J. Hutter, M. Iannuzzi, F. Schiffmann, and J. Vandevondele, "Cp2k: Atomistic Simulations of Condensed Matter Systems," *Wiley Interdisciplinary Reviews: Computational Molecular Science 4*, no. 1 (2014): 15–25, https://doi.org/10.1002/wcms.1159.
2. J. Vandevondele, M. Krack, F. Mohamed, M. Parrinello, T. Chassaing, and J. Hutter, "Quickstep: Fast and Accurate Density Functional Calculations Using a Mixed Gaussian and Plane Waves Approach," *Computer Physics Communications 167*, no. 2 (2005): 103–128, https://doi.org/10.1016/j.cpc.2004.12.014.
3. E. Acuna-Yeomans, P. J. Goosen, J. J. Gutierrez-Sevillano, D. Dubbeldam, and S. Calero, "Effect of Water Loading on the Stability of Pristine and Defective UiO-66," *Journal of Materials Chemistry A 12*, no. 37 (2024): 25233–25243, https://doi.org/10.1039/d4ta04252b.
4. J. P. Perdew, K. Burke, and M. Ernzerhof, "Generalized Gradient Approximation Made Simple," *Generalized Gradient Approximation Made Simple*, 1996.
5. S. Goedecker, M. Teter, and J. Hutter, "Separable Dual-Space Gaussian Pseudopotentials," *Separable dual-space Gaussian pseudopotentials*, 1996.
6. C. Huang, M. Dontigny, K. Zaghib, and P. S. Grant, "Low-Tortuosity and Graded Lithium Ion Battery Cathodes by Ice Templating," *Journal of Materials Chemistry A 7*, no. 37 (2019): 21421–21431, https://doi.org/10.1039/c9ta07269a.
7. C. Ma, Y. Zhang, Y. Feng, et al., "Engineering Fe–N Coordination Structures for Fast Redox Conversion in Lithium–Sulfur Batteries," *Advanced Materials 33*, no. 30 (2021), https://doi.org/10.1002/adma.202100171.
8. Y. Zhang, J. Liu, J. Wang, et al., "Engineering Oversaturated Fe-N5 Multifunctional Catalytic Sites for Durable Lithium-Sulfur Batteries," *Angewandte Chemie - International Edition 60*, no. 51 (2021): 26622–26629, https://doi.org/10.1002/anie.202108882.
9. C. Dong, C. Zhou, M. Wu, et al., "Boosting Bi-Directional Redox of Sulfur with Dual Metal Single Atom Pairs in Carbon Spheres Toward High-Rate and Long-Cycling Lithium–Sulfur Battery," *Advanced Energy Materials 13*, no. 30 (2023),

https://doi.org/10.1002/aenm.202301505.
10. S. Zhang, X. Ao, J. Huang, et al., "Isolated Single-Atom Ni-N5Catalytic Site in Hollow Porous Carbon Capsules for Efficient Lithium-Sulfur Batteries," *Nano Letters 21*, no. 22 (2021): 9691–9698, https://doi.org/10.1021/acs.nanolett.1c03499.
11. X. Meng, X. Liu, X. Fan, et al., "Single-Atom Catalyst Aggregates: Size-Matching Is Critical to Electrocatalytic Performance in Sulfur Cathodes," *Advanced Science 9*, no. 3 (2022), https://doi.org/10.1002/advs.202103773.
12. Y. Li, P. Zhou, H. Li, et al., "A Freestanding Flexible Single-Atom Cobalt-Based Multifunctional Interlayer toward Reversible and Durable Lithium-Sulfur Batteries," *Small Methods 4*, no. 3 (2020), https://doi.org/10.1002/smtd.201900701.
13. B. Q. Li, L. Kong, C. X. Zhao, et al., "Expediting Redox Kinetics of Sulfur Species by Atomic-Scale Electrocatalysts in Lithium–Sulfur Batteries," *InfoMat 1*, no. 4 (2019): 533–541, https://doi.org/10.1002/inf2.12056.
14. C. Wang, H. Song, C. Yu, et al., "Iron Single-Atom Catalyst Anchored on Nitrogen-Rich MOF-Derived Carbon Nanocage to Accelerate Polysulfide Redox Conversion for Lithium Sulfur Batteries," *Journal of Materials Chemistry A 8*, no. 6 (2020): 3421–3430, https://doi.org/10.1039/c9ta11680j.
15. X. Tu, L. Zhu, L. Shang, et al., "Two-Phase Tandem Catalysis With Interfacial Synergy in MoNix/Mo2C Heterostructures for Stepwise Sulfur Conversion," *Advanced Functional Materials* (2026), https://doi.org/10.1002/adfm.76702.
16. X. Men, T. Deng, J. Che, and J. Wang, "Accelerating Sulfur Conversion Kinetics via CoS2–MgS Heterostructure for Lithium Sulfur Batteries," *Journal of Materials Chemistry A 12*, no. 46 (2024): 31972–31981, https://doi.org/10.1039/d4ta06086e.
17. X. Li, Y. Zuo, Y. Zhang, et al., "Controllable Sulfurization of MXenes to In-Plane Multi-Heterostructures for Efficient Sulfur Redox Kinetics," *Advanced Energy Materials 14*, no. 9 (2024), https://doi.org/10.1002/aenm.202303389.
